# Supplementary material for: Adults with more severe psychopathy in the community show increased social discounting
Source: Commun Psychol. 2025 Nov 26;3:175. doi: 10.1038/s44271-025-00353-z (PMC12658011; doi:10.1038/s44271-025-00353-z)
Supplement: Supplementary file 2 — Supplementary Information [file 44271_2025_353_MOESM2_ESM.pdf]

Adults with More Severe Psychopathy in the Community Show Increased Social Discounting

Supplementary Information

Naomi Nero[1]

Marla Dressel[1] Paige Amormino[1] Paige Freeburg[2] Melinda Somers[1]

Lindsay Poplinski[3] Katie Duhamel[3] Viviana Alvarez-Toro[3]

Abigail Marsh[1]

[1] Department of Psychology, Georgetown University, Washington DC, United States of America

[2] Department of Psychology, Yale University, New Haven, Connecticut, United States of America

[3] St. Elizabeths Hospital, DC Department of Behavioral Health, Washington DC, United States of America

Author Note

Naomi Nero https://orcid.org/0009-0004-7941-1482

Marla Dressel https://orcid.org/0009-0001-6956-4985

Paige Amormino https://orcid.org/0000-0003-2125-1047

Paige Freeburg https://orcid.org/0000-0001-6229-8314

Melinda Somers https://orcid.org/0009-0000-8658-3304

Lindsay Poplinski https://orcid.org/0009-0004-7538-662X

Katie Duhamel https://orcid.org/0009-0003-4242-571X

Viviana Alvarez-Toro https://orcid.org/0009-0006-1796-6540

Abigail A. Marsh https://orcid.org/0000-0001-5635-181X

Correspondence concerning this article should be addressed to Naomi Nero, Department of Psychology, Georgetown University, White Gravenor Hall, Washington, DC 20057, United States. Email: nn444@georgetown.edu

Supplementary Analyses

Reliability and robustness

We examined the reliability of responses by conducting additional Cronbach's alpha analyses within each recruitment method group. After excluding participants for failing two or more attention checks, participants recruited through the online psychopathy resource had high reliability in their total TriPM score and subscales (TriPM total: α = .83, 95% CI [.79 0.85]; TriPM Meanness: α = 0.82 [0.79, 0.85]; TriPM Disinhibition: α = 0.80, 95% CI [0.77, 0.83]; TriPM Boldness: α = 0.82, 95% CI [0.79, 0.85], along with their total STAB score (STAB: α = .91, 95% CI [0.89, 0.93]). Participants recruited through CloudResearch also had high reliability in their total TriPM, TriPM subscales, and total STAB score (TriPM total: α = .89, 95% CI [.88 0.90]; TriPM Meanness: α = 0.89, 95% CI [0.888, 0.91]; TriPM Disinhibition: a = 0.89, 95% CI [0.88, 0.91]; TriPM Boldness: α = 0.88, 95% CI [0.86, 0.89]; STAB: α = .95, 95% CI [0.94, 0.96]). This suggests strong internal consistency regardless of recruitment source.

Lastly, following recently generated recommendations for maximizing the quality of online data, in the beginning of the survey, we included a commitment request question (Geisen, 2022; Hibben et al., 2022): "Do you commit to providing thoughtful answers to the questions in this survey". Participants could respond with "Yes", "I can't promise", or "No", with participants excluded for selecting "No". No participants in either participant group selected "No". We compared the proportion of participants who responded "I can't promise" versus "Yes" across recruitment sources (1.7% from psychopathy online platform; 0.28% from CloudResearch) and found no group differences ($\chi^2(1) = 2.34$, p = .13).

To assess the robustness of our results in the main manuscript, we conducted two robustness checks: 10-fold cross-validation and propensity score matching. We conducted 10-fold cross-validation using the caret package in R (Kuhn, 2008). A linear regression model predicting logk were trained and tested across folds, including covariates (age, gender, income, fluid intelligence). Results showed that the high psychopathy group had a significantly higher social discounting, *B* = 0.38, p < .001 (Table S2). Model performance was stable across iterations (average root mean square error = 1.84; SD = 0.08), confirming the robustness of the group effect. This relationship remained significant when assessing psychopathy continuously (B = 0.40, p < .001, RMSE = 1.82, SD = 0.07, Table S2), and when examining subscales, only meanness was significantly associated with logk (B = 0.41, p < .001, RMSE = 1.80, SD = 0.08, Table S2).

Additionally, propensity score matching was used to estimate the group differences in logk. Logistic regression was estimated based on gender (Male or female/other), age, household income, and fluid intelligence. One-to-one nearest neighbor matching was used and all participants in the high psychopathy group were matched to a participant in the control group using a caliper of 0.20. This resulted in a sample size of 476, with 238 high psychopathy participants matched to 238 control participants (Table S3). The matched data had good balance between the control and high psychopathy group, with all standardized mean differences below 0.1. We then used a hyperbolic model to examine the group differences in the social discounting curve and individual logk values were calculated for each participant. In a linear regression model, there were significant group differences in logk with the high psychopathy group having

a significantly steeper discounting curve, *B* = 0.36, *p* < .001, 95% CI [0.27, 0.44] (Table S4). Similar results were observed when the relationship between discounting (logk) and psychopathy was assessed as a continuous measure across all participants, with social discounting again increasing as psychopathy increased, *B* = 0.36, *p* < .001, 95% CI [0.27, 0.44] (Table S4). In a multiple linear regression including meanness, disinhibition, and boldness subscale scores as predictors of logk. Meanness was the only subscale that predicted logk, *B* = .41, *p* < .001, 95% CI [0.26, 0.56] (Table S4). The high psychopathy group had significantly higher antisocial behavior (*B* = 0.68, p < .001, 95% CI [0.61, 0.74]) and significantly higher total psychopathy scores, B = 0.87, p < .001, 95% CI [0.82, 0.91]).

Spline model

In addition to the hyperbolic model assessing group differences in logk, from which we extract individual logk values used in the main manuscript analyses, we also implemented a piecewise linear spline regression approach to flexibly model psychopathy as a continuous predictor while accounting for the clinically relevant thresholds that separate the groups. Using gender specific knots based on established TriPM cutoffs for clinically high psychopathy (Berluti et al., 2025; Male = 105; Female/Other = 91), we constructed separate B-spline basis functions for males and females. After extracting individual logk values from the hyperbolic model presented in the manuscript, spline models were used to estimate basis function values for each participant based on their TriPM score, utilizing a single knot at the cutoff point for their gender. These basis values were then put in a multiple regression model with age, gender, fluid intelligence, and income as covariates. The resulting model revealed that psychopathic traits was associated with increased logk for females below the clinical cutoff (B = 0.21, 95% CI [0.05, 0.36], p = .01) but not for males below the clinical cutoff (B = 0.08, 95% CI [-0.08, 0.24], p = .32) (Table S1). In contrast, psychopathic traits was robustly associated with increased logk above the cutoff in both females (B = 0.38, 95% CI [0.31, 0.46], p < .001) and males (B = 0.23, 95% CI [0.16, 0.31], p < .001) (Table S1). The spline analyses reinforce the findings from our primary models, providing additional confidence in the robustness of our conclusions.

AUC results

In addition to examining the relationship with logk, we ran analyses using the area under the hyperbolic curve (AUC), which revealed very similar results. A bivariate association between psychopathy group and AUC was observed, *t*(692.49) = 8.42, *p* < .001, Cohen’s d=0.62, which persisted after controlling for age, gender, income, and fluid intelligence, *B* = -0.27, *p* < .001, 95% CI [-0.35, -0.19] (Table S18). Similar results were observed when the relationship between AUC and psychopathy was assessed as a continuous measure, *B* = -0.26, *p* < .001, 95% CI [-.34, -.18] (Table S19; Figure S3). To identify whether the variance associated with one or more subscales of psychopathy was driving this association, we conducted a multiple linear regression including meanness, disinhibition, and boldness subscale scores as predictors of AUC. Meanness was the only subscale that predicted AUC, *B* = -.43, *p* < .001, 95% CI [-0.56, -0.30] (Table S20), and the bivariate association between meanness and AUC was also significant, *r*(713) = -0.33, *p* < .001, 95% CI [-0.39, -0.27].

There was a significant bivariate association between AUC and antisocial behavior, *r*(713) = -0.24, p<.001, 95% CI [-0.31, -0.17], which persisted when controlling for covariates, *B*= -0.20, *p*

< .001, 95% CI [-0.27, -0.13], *F*(5,709) = 24.25. Mediation models were run to see if AUC explained group differences in antisocial behavior. AUC did not mediate the association between group and antisocial behavior (total effect *p* < .001, direct effect *p* < .001, indirect effect *p* = .51; Table S21), or when considering psychopathy as a continuous variable (total effect *p* < .001, direct effect *p* < .001, indirect effect *p* = .53; Table S22). AUC did not mediate the relationship between psychopathy and antisocial behavior within the high psychopathy group (total effect: *p* < .001, direct effect: *p* < .001, indirect effect: *p* = 0.11) or within the control group (total effect: *p* < .001, direct effect: *p* < .001, indirect effect: *p* = .91).

We observed a significant moderating effect such that as AUC increased, antisocial behavior decreased in the high psychopathy group, but there was no association between AUC and antisocial behavior in controls, Psychopathy Group x AUC *B* = -0.07, *p* = .02, 95% CI [-0.13, -0.01] (Table S23; Figure S4). However, there was no moderating effect when examining psychopathy as a continuous measure, *B* = 0.01, *p* = .79, 95% CI [-0.04, 0.06] (Table S24).

Lastly, regression models were run to see if age moderated the relationship between psychopathic traits and AUC. Similar to logk, there was no significant interaction when psychopathy was treated either dichotomously, *B* = -0.04, *p* = .29, 95% CI [-0.13, 0.04] (Table S25), or continuously, *B* = -0.05, *p* = .23, 95% CI [-0.13, 0.03] (Table S26).

Analyses dropping participants outside of their recruited groups' range

We reran analyses excluding participants recruited for the high psychopathy group whose summed TriPM score was not above the cutoff and excluding participants recruited for the control group whose summed TriPM score was above the cutoff. This was to verify that results stayed consistent as when we reassigned group membership based on TriPM score. This resulted in 627 total participants (*n* = 349 control; *n* = 278 high psychopathy; Table S27). The mean psychopathy score for participants in our high psychopathy group was 122.78 (*SD* = 16.93, range = 91-165), which was significantly higher than the mean psychopathy score in the control group (*M* = 51.85, *SD* =17.65, range = 19-104, *t*(603.9) = -51.15, *p* < .001, Cohen's d = -4.09). Groups did not differ in gender composition or race/ethnicity. However, the control group was older, *t*(624.71) = 12.42, *p* < .001, Cohen's d = 0.97; more educated, *t*(577.1) = 6.77, *p* < .001, Cohen's d =.55; and had higher income, *t*(552.65) = -2.18, *p* = .03, Cohen's d = -0.18. Age, gender (female/other as the reference), income, and fluid intelligence were included as covariates in all the following analyses. (14 participants who reported not knowing their household income were recoded as being in the mean income bracket of the full sample.) Group differences in TriPM score persisted when controlling for age, gender, fluid intelligence, and income, *B* = 0.88, 95% CI [0.84, 0.91], *p* < .001, *F*(5,621) = 560.1. Consistent with their psychopathy scores, high-psychopathy participants also reported significantly more antisocial behavior (*M* = 95.02, *SD* = 18.24, range = 52-155) than controls (*M* = 57.05, *SD* = 18.01, range = 32-138), *t*(590.76) = -26.04, *p* < .001, Cohen's d = -2.10 (Table S28). Group differences persisted when controlling for age, gender, fluid intelligence, and income, *B* = 0.71, 95% CI [0.65, 0.77], *p* < .001, *F*(5,621) = 141.3.

Before analyzing the difference in discounting across social distances in both groups, we checked to see if the hyperbolic model was the best fit model by examining AIC values. The

hyperbolic model yielded the lowest AIC value (40406.3), indicating a superior fit compared to the exponential (AIC: 42889.3) and linear (AIC: 42338.8) models. A hyperbolic discounting curve was thus modeled to estimate discounting rates for each participant.

While controlling for age, gender, fluid intelligence, and income the hyperbolic model indicated significant increases in social discounting (higher logk) among high-psychopathy participants compared to controls, b = 1.63, $p < .001$, 95% CI [1.27, 1.98], $t = 8.95$ (Table S30).

Individual logk values were extracted for each participant. A bivariate association between psychopathy group and logk was also observed, $t(594.78) = -12.32$, $p < .001$, Cohen’s d = -0.99, which persisted after controlling for age, gender, income, and fluid intelligence, $B = 0.40$, $p < .001$, 95% CI [0.33, 0.48] (Table S31), indicating that high-psychopathy participants show a significantly steeper hyperbolic decay in generosity as social distance increases relative to controls. No main effects of gender, income, or fluid intelligence were observed. However, a main effect of age was observed, such that social discounting decreased as age increased, $B = -0.10$, $p = .01$, 95% CI [-0.18, -0.02]. Similar results were observed when the relationship between discounting (logk) and psychopathy was assessed as a continuous measure, with social discounting again increasing as psychopathy increased, $B = 0.41$, $p < .001$, 95% CI [0.33, 0.49] (Table S32), with the bivariate association also being significant, $r(625) = 0.44$, $p < .001$, 95% CI [0.38, 0.50].

Similar results were observed with the relationship between AUC and psychopathy. A bivariate association between psychopathy group and AUC was observed, $t(624.47) = 8.28$, $p < .001$, Cohen’s d=0.65, as well as a significant bivariate association between psychopathic traits and AUC, $r(625) = -.29$, $p < .001$, 95% CI [-0.36, -0.21]. Both of these associations persisted after controlling for age, gender, fluid intelligence, and income (dichotomous: $B = -0.28$, $p < .001$, 95% CI [-0.37, -0.19], Table S33; continuous: $B = -0.26$, $p < .001$, 95% CI [-.34, -.17], Table S34), indicating a negative relationship between psychopathy and AUC.

To identify whether the variance associated with one or more subscales of psychopathy was driving these associations between psychopathy and logk/AUC, we conducted a multiple linear regression including meanness, disinhibition, and boldness subscale scores as predictors of logk and AUC, respectively. Only the meanness subscale significantly predicted logk while controlling for the other subscales ($B =.44$, $p < .001$, 95% CI [0.30, 0.58], Table S35). The bivariate association between meanness and logk was significant ($r(625) = 0.47$, $p < .001$, 95% CI [0.41, 0.52]). Meanness was the only subscale that predicted AUC, $B = -.42$, $p < .001$, 95% CI [-0.57, -0.27] (Table S36), and the bivariate association between meanness and AUC was also significant, $r(625) = -.33$, $p < .001$, 95% CI [-0.39, -0.26].

We found a bivariate association between logk and antisocial behavior across the full sample, $r(625) = .37$, $p < .001$, 95% CI [0.30, 0.44], which remained statistically significant after controlling for age, gender, fluid intelligence, and income ($B = 0.30$, 95% CI [0.23, 0.37], $p < .001$, $F(5,621) = 33.60$). Similar results were found for the relationship between AUC and antisocial behavior, $r(625) = -.24$, $p < .001$, 95% CI [-0.31, -0.16], which remained statistically significant after controlling for demographic variables, $B = -.18$, 95% CI [-0.26, -0.11], $p < .001$, $F(5,621) = 23.94$.

Mediation analysis completed using the mediation package in R (Tingley, 2014) found that social discounting (logk) did not mediate group differences in antisocial behavior (total effect: $p < .001$, direct effect: $p < .001$, indirect effect: $p = 0.068$; Table S37). Logk also did not mediate the relationship between psychopathy, as a continuous variable, and antisocial behavior (total effect: $p < .001$, direct effect: $p < .001$, indirect effect: $p = .68$; Table S38). AUC did not mediate group differences in antisocial behavior (total effect: $p < .001$, direct effect: $p<.001$, indirect effect: $p = .58$; Table S39) or the relationship between psychopathic traits and antisocial behavior (total effect: $p < .001$, direct effect: $p < .001$, indirect effect: $p = .69$; Table S40).

We further investigated whether discounting mediated the relationship between psychopathic traits and antisocial behavior within each group. This revealed that both logk and AUC partially mediated the relationship between psychopathic traits and antisocial behavior within the high psychopathy group (logk - total effect: $p < .001$, direct effect: $p < .001$, indirect effect: $p = .004$, proportion mediated: b = 0.07, $p = 0.004$; AUC - total effect: $p < .001$, direct effect: $p < .001$, indirect effect: $p = 0.012$, proportion mediated: b = 0.03, $p = 0.012$). However, there was not a significant mediation in the control group (logk - total effect: $p < .001$, direct effect: $p < .001$, indirect effect: $p = .81$; AUC - total effect: $p < .001$, direct effect: $p < .001$, indirect effect: $p = .44$)

We also observed significant moderating relationships between discounting (logk) and psychopathy group in predicting antisocial behavior, $B = 0.13$, $p < .001$, 95% CI [0.07, 0.19] (Table S41), such that as social discounting (logK) increases, antisocial behavior increases at a higher rate in the high-psychopathy group relative to controls. However, this was not seen when psychopathy was measured continuously, $B = 0.02$, $p = .37$, 95% CI [-0.03, -0.7] (Table S42). AUC did not moderate group differences in antisocial behavior, $B = -0.01$, $p = .75$, 95% CI [-0.06, 0.04], Table S43. However, AUC did significantly moderate the relationship between psychopathic traits and antisocial behavior ($B = -0.11$, $p < .001$, 95% CI [-0.17, -0.06], Table S44)

We replicated the negative association between antisocial behavior and age across the full sample, $B = -.34$, $p < .001$, 95% CI [-.42, -.27], $F(4,622) = 23.07$). However, when examining if there were group differences in the relationship between age and antisocial behavior, the high psychopathy group exhibited a *positive* association between age and antisocial behavior, whereas the opposite association was observed in controls when controlling for gender, income, and fluid intelligence (Age x Group $B = .10$, $p = .002$, 95% CI [0.04, 0.16], $F(6,620) = 121$). We therefore conducted a multiple regression analysis to determine whether age moderates the relationship between psychopathic traits and discounting. Results indicated that age did not moderate the relationship between psychopathy (treated either dichotomously or continuously) and discounting indexed by logk (dichotomous: $B = 0.04$, $p = .32$, 95% CI [-0.04, 0.13], Table S45; continuously: $B = 0.03$, $p = .40$, 95% CI [-0.05, 0.11], Table S46). There was also no moderating effect of age on the relationship between psychopathy and AUC when modeling psychopathic traits dichotomously, $B = -0.05$, $p = .25$, 95% CI [-0.14, 0.04], Table S46, or continuously, $B = -0.06$, $p = .20$, 95% CI [-0.14, 0.03], Table S48.

Supplementary Figures/Tables

The following are supplemental tables for the logk analyses where participants in the high psychopathy group who were below the 95th percentile cutoff for their gender were transferred to controls and controls above the cutoff were transferred to the high psychopathy group (as reported in the main text).

Table S1

Predicting logk Above and Below TriPM Cutoffs Using Spline Regression Model

Variables	*b (se)*	CI	std. B *(se)*	CI	*p*
(Intercept)	-2.66 (0.44)	-3.52, -1.80	0.00 (0.03)	-0.06, 0.06	**<.001**
Male Below Cutoff	0.50 (0.41)	-0.30, 1.30	0.10 (0.08)	-0.06, 0.26	.221
Male Above Cutoff	2.79 (0.44)	1.93, 3.65	0.24 (0.04)	0.17, 0.32	**<.001**
Female Below Cutoff	1.05 (0.37)	0.33, 1.77	0.23 (0.08)	0.07, 0.38	**.005**
Female Above Cutoff	4.17 (0.40)	3.38, 4.96	0.39 (0.04)	0.32, 0.46	**<.001**
Age	-0.02 (0.01)	-0.03, -0.01	-0.12 (0.04)	-0.19, -0.04	**.002**
Income	-0.06 (0.03)	-0.13, 0.00	-0.07 (0.03)	-0.13, 0.00	.05
Gender (Male > Female/Other)	0.78 (0.45)	-0.11, 1.67	0.19 (0.11)	-0.03, 0.41	.086
Fluid Intelligence	0.09 (0.03)	0.02, 0.16	0.09 (0.03)	0.02, 0.16	**.009**
	$F(8,706) = 26.31$, Adjusted $R^2 = 0.22$				

Note. With the full dataset presented in the main manuscript, we predicted logk using a piecewise linear spline regression model, with gender-specific knots according to the TriPM cutoff for clinical levels of psychopathy (Male = 105, Female/Other = 91). $p < .05$ in bold.

Table S2

Psychopathy Predicting logk Using 10-fold Cross Validation

Variables	*b*	std. B	*p*
(Intercept)	-2.20	-	**< .001**
High Psychopathy > Controls	1.56	0.38	**< .001**
Age	-0.02	-0.12	**< .001**
Gender (Male > Female and Other)	0.12	0.03	.37
Income	-0.07	-0.07	.046
Fluid Intelligence	0.09	0.09	.005
Adjusted R^2 = 0.191			
F(5, 709) = 34.62, p < .001			
RMSE = 1.83, SD = 0.08			
(Intercept)	-3.41		**< .001**
Psychopathy (TriPM)	0.02	0.40	**< .001**
Age	-0.02	-0.10	**.005**
Gender (Male > Female and Other)	-0.04	-0.01	.76
Income	-0.07	-0.08	**.02**
Fluid Intelligence	0.1	0.1	**.003**
Adjusted R^2 = 0.21			
F(5, 709) = 36.86, p < .001			
RMSE = 1.82, SD = 0.07			
(Intercept)	-3.05		**< .001**
Meanness (TriPM)	0.05	0.41	**< .001**
Disinhibition (TriPM)	0.006	0.04	.40
Boldness (TriPM)	-0.005	-0.03	.51
Age	-0.01	-0.08	**.039**
Gender (Male > Female and Other)	-0.03	-0.01	.82
Income	-0.06	-0.06	.08
Fluid Intelligence	0.1	0.1	**.003**
Adjusted R^2 = 0.23			
F(7,707) = 29.8, p < .001			
RMSE = 1.80, SD = 0.08			

Note. RMSE = root mean squared error. *p* < .05 in bold.

Table S3

	Unmatched			Matched		
	High Psychopathy Mean	Control Mean	Std. Mean Difference	High Psychopathy Mean	Control Mean	Std. Mean Difference
Propensity Score (Distance)	0.51	0.33	0.97	0.47	0.45	0.11
Male	0.43	0.45	-0.04	0.44	0.43	0.02
Age	31.49	40.2	-0.98	33.07	33.9	-0.09
Income	3.74	3.5	0.11	3.74	3.62	0.06
Fluid Intelligence	5.16	5.57	-0.21	5.32	5.28	0.02

Note. The original sample size (unmatched) included 427 controls and 238 high psychopathy participants. 238 controls were matched with the 238 high psychopathy participants, leaving 189 controls unmatched. This resulted in a matched sample of 476.

Table S4

Psychopathy Predicting logk in the Matched Sample

	b (se)	CI	std. B *(se)*	CI	*p*
(Intercept)	-2.41 (0.15)	-2.70, -2.12	0.00 (0.04)	-0.08, 0.08	**<.001**
High Psychopathy > Controls	1.76 (0.21)	1.35, 2.18	0.36 (0.04)	0.27, 0.44	**<.001**
Adjusted R^2 = 0.126					
F(1,474) = 69.95, p< .001					
(Intercept)	-3.68 (0.28)	-4.22, -3.13	0.00 (0.04)	-0.08, 0.08	**<.001**
Psychopathy (TriPM)	0.02 (0.00)	0.02, 0.03	0.36 (0.04)	0.27, 0.44	**<.001**
Adjusted R^2 = 0.127					
F(1,474) = 70.08, p < .001					
(Intercept)	-2.87 (0.36)	-3.57, -2.17	0.00 (0.04)	-0.08, 0.08	**<.001**
Meanness (TriPM)	0.06 (0.01)	0.04, 0.09	0.41 (0.08)	0.26, 0.56	**<.001**
Disinhibition (TriPM)	0.00 (0.01)	-0.02, 0.02	0.01 (0.07)	-0.12, 0.14	.901
Boldness (TriPM)	-0.01 (0.01)	-0.03, 0.01	-0.05 (0.05)	-0.15, 0.05	.317
Adjusted R^2 = 0.149					
F(3,472) = 28.71, p < .001					

Note. p < .05 in bold.

Figure S1

Social Discounting Task

Remember you made a list of the 100 people closest to you in the world ranging from your dearest friend or relative at #1 to a mere acquaintance or stranger at #100. You will now be making choices for the #1 person on the list, XXX.

Now imagine the following choice between an amount of money for you and an amount for the #1 person on the list, XXX.

$155 for you alone	$75 for you and $75 for the #1 person on the list
○	○

Now imagine the following choice between an amount of money for you and an amount for the #1 person on the list, XXX.

$145 for you alone	$75 for you and $75 for the #1 person on the list
○	○

Now imagine the following choice between an amount of money for you and an amount for the #1 person on the list, XXX.

$135 for you alone	$75 for you and $75 for the #1 person on the list
○	○

Now imagine the following choice between an amount of money for you and an amount for the #1 person on the list, XXX.

$125 for you alone	$75 for you and $75 for the #1 person on the list
○	○

•
•
•

Now imagine the following choice between an amount of money for you and an amount for the #1 person on the list, XXX.

$75 for you alone	$75 for you and $75 for the #1 person on the list
○	○

Note. An example of one block of the social discounting task where the participant makes a series of monetary decisions between them and their N = 1 on the list they previously provided. The names of the social distances were inserted into each question. This is then repeated for the 6 other social distances (N = 1, 2, 5, 10, 20 50, 100) (Jones & Rachlin, 2006).

Table S5

Descriptive Statistics of Main Variables

	Full Sample				Control (N=427)				High Psychopathy (N=278)			
Variable	*M* (*SD*)	Range	Skewness	Kurtosis	*M* (*SD*)	Range	Skewness	Kurtosis	*M* (*SD*)	Range	Skewness	Kurtosis
Total Psychopathy (TriPM)	82.60 (37.59)	19 - 165	0.21	1.80	55.86 (19.90)	19 - 104	0.44	2.33	122.25 (16.92)	91 - 165	0.31	2.55
Meanness	23.60 (16.64)	0 - 57	0.24	1.73	12.12 (9.34)	0 - 40	0.77	2.78	40.62 (8.52)	17 - 57	-0.23	2.38
Boldness	33.53 (11.81)	2 - 57	-0.29	2.47	28.04 (10.52)	2 - 53	-0.11	2.65	41.67 (8.44)	14 - 57	-0.54	2.77
Disinhibition	25.47 (15.74)	0 - 58	0.16	1.81	15.70 (10.76)	0 - 50	0.70	2.76	39.96 (9.60)	8 - 58	-0.44	2.68
STAB	73.50 (25.60)	32 - 155	0.49	2.53	58.81 (18.07)	32 - 138	1.23	5.23	95.27 (18.67)	44 - 155	0.37	3.11
Fluid IQ	5.41 (2.05)	0 - 13	0.72	3.42	5.57 (2.09)	1 - 13	0.82	3.50	5.16 (1.96)	0 - 10	0.50	3.00
AUC	0.30 (0.35)	-0.06- 0.99	0.87	2.34	0.38 (0.36)	-0.06 - 0.99	0.56	1.80	0.17 (0.29)	-0.06 - 0.99	1.52	4.32
logk	-2.04 (2.03)	-5.77 - 1.81	-0.15	2.27	-2.72 (1.85)	-5.77 - 1.36	-0.01	2.30	-1.03 (1.86)	-5.46 - 1.81	-0.56	2.80

Note. Fluid Intelligence exhibited slight kurtosis. However, log transformation did not improve model performance, so untransformed values were retained in the final analyses.

Table S6

Group Differences in Antisocial Behavior

Variable	*b (se)*	CI	Std. B *(se)*	Std. CI	*p*
(Intercept)	66.68 (3.48)	59.85 , 73.50	0.00 (0.03)	-0.05, 0.05	**<.001**
High Psychopathy > Control	35.62 (1.51)	32.65, 38.59	0.68 (0.03)	0.63, 0.74	**<.001**
Age	-0.10 (0.06)	-0.23, 0.02	-0.05 (0.03)	-0.10, 0.01	.11
Gender (Male > Female/Other)	3.31 (1.38)	0.60, 6.01	0.06 (0.03)	0.01, 0.12	**.02**
Income	-0.76 (0.33)	-1.41, -0.11	-0.06 (0.03)	-0.12, -0.01	**.02**
Fluid Intelligence	-0.45 (0.34)	-1.12, 0.22	-0.04 (0.03)	-0.09, 0.02	.18
	F(5,709) = 142, p < .001, Adjusted R^2 = 0.497				

Note. p < .05 in bold

Table S7. *Group Differences in Likelihood of Committing at Least 1 Crime*

Variable	Odds Ratio	CI	*p*
(Intercept)	0.15	0.06, 0.35	**<.001**
High Psychopathy > Control	12.99	8.48, 20.39	**<.001**
Age	1.03	1.01, 1.05	**.001**
Gender (Male > Female/Other)	1.15	0.81, 1.63	.42
Income	0.94	0.86, 1.02	.12
Fluid Intelligence	1.11	1.02, 1.22	**.01**
AIC = 797.37			
R^2 = 0.224			

Table S8. *Group Differences in Likelihood of Being Charged with at Least 1 Crime*

Variable	Odds Ratio	CI	*p*
(Intercept)	0.09	0.04, 0.23	**<.001**
High Psychopathy > Control	3.87	2.61, 5.79	**<.001**
Age	1.04	1.02, 1.06	**<.001**
Gender (Male > Female/Other)	1.57	1.11, 2.24	**.01**
Income	0.85	0.77, 0.93	**<.001**
Fluid Intelligence	0.94	0.86, 1.03	.19
AIC = 782.8			
R^2 = 0.093			

Table S9. *Group Differences in Likelihood of Being Convicted of at Least 1 Crime*

Variable	Odds Ratio	CI	*p*
(Intercept)	0.13	0.05, 0.34	**<.001**
High Psychopathy > Control	3.09	2.06, 4.67	**<.001**
Age	1.03	1.01, 1.05	**.001**
Gender (Male > Female/Other)	1.53	1.06, 2.21	**.02**
Income	0.87	0.79, 0.96	**.004**
Fluid Intelligence	0.91	0.83, 1.00	.06
AIC = 736.13			
R^2 = 0.067			

Table S10

Hyperbolic Model Predicting Maximum Amount Willing to Forgo for each Social Distance in High Psychopathy and Control Group

Variable	*b*	*SE*	CI	*p*
(Intercept)	86.22	0.72	84.80, 87.63	**<.001**
logk, mean discounting rate for controls	-2.2	0.39	-2.97, -1.43	**<.001**
High Psychopathy > Controls	1.56	0.17	1.23, 1.90	**<.001**
Age	-0.02	0.01	-0.04, -0.01	**.003**
Gender (Male > Female/Other)	0.12	0.16	-0.18, 0.43	.43
Household Income	-0.07	0.04	-0.14, 0.01	.08
Fluid Intelligence	0.1	0.04	0.02, 0.17	**.01**

Note. $p < .05$ in bold

Figure S2

Psychopathic Traits Predicting Social Discounting Rate (logk)

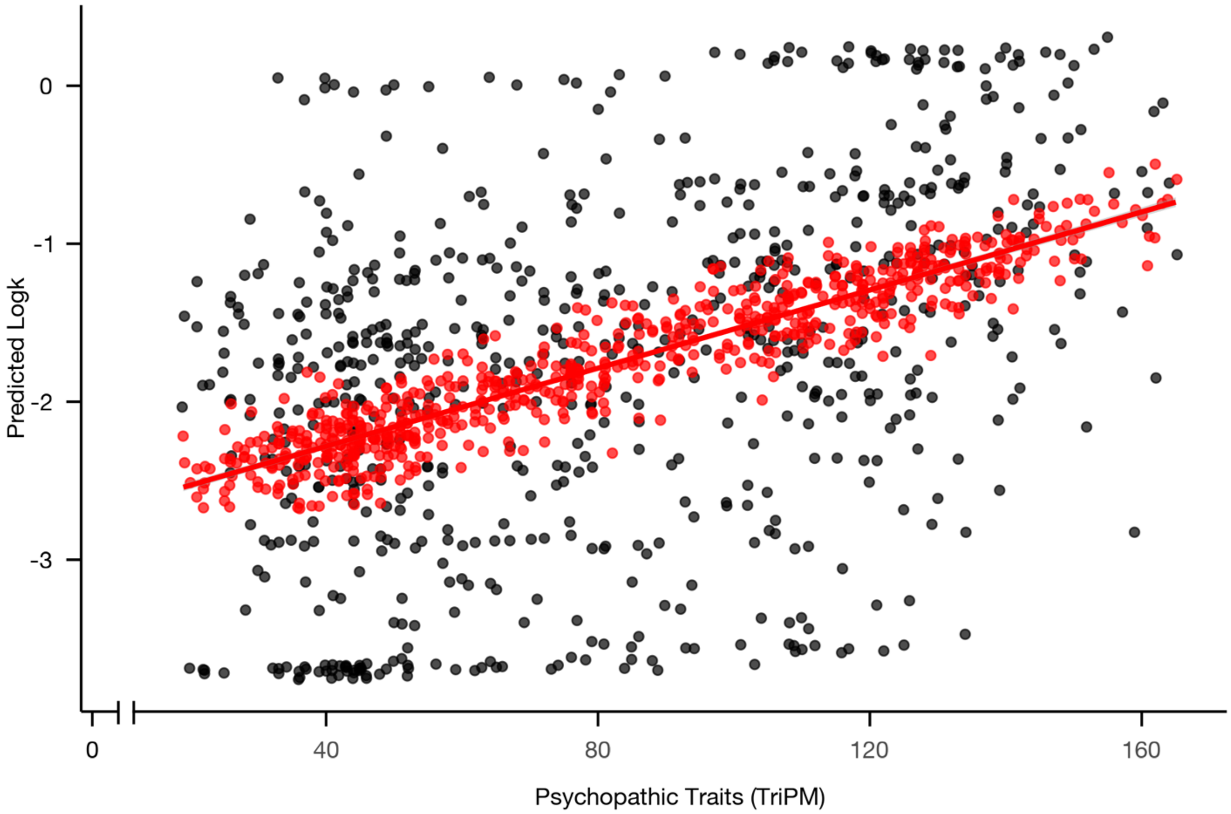

Note. The positive association between psychopathic traits and logk with black representing raw logk values, and red representing predict logk values from the linear regression model controlling for age, gender, and income.

Table S11

Boldness, Meanness, and Disinhibition Predicting Social Discounting Rate (logK)

Variable	*b (se)*	CI	Std. B *(se)*	Std. CI	*p*
(Intercept)	-3.05 (0.41)	-3.86, -2.24	0.00 (0.03)	-0.06, 0.06	**<.001**
Meanness (TriPM)	0.05 (0.01)	0.04, 0.07	0.41 (0.06)	0.29, 0.54	**<.001**
Disinhibition (TriPM)	0.01 (0.01)	-0.01, 0.02	0.04 (0.05)	-0.06, 0.15	.41
Boldness (TriPM)	0.00 (0.01)	-0.02, 0.01	-0.03 (0.04)	-0.11, 0.05	.51
Age	-0.01 (0.01)	-0.03, -0.001	-0.08 (0.04)	-0.15, -0.004	**.04**
Male > Female/Other	-0.03 (0.14)	-0.30, 0.24	-0.01 (0.03)	-0.07, 0.06	.82
Income	-0.06 (0.03)	-0.13, 0.01	-0.06 (0.03)	-0.13, 0.01	.08
Fluid Intelligence	0.10 (0.03)	0.04, 0.17	0.10 (0.03)	0.04, 0.17	**.003**
	$F(7,707) = 29.80$, $p < .001$, Adjusted $R^2 = 0.22$				

Note. p < .05 in bold

Table S12

logk Mediating the Relationship Between Psychopathy Group and Antisocial Behavior

Variable	logk (M)				STAB (Y)			
	path	*b (se)*	Std. B (se)	*p*	path	*b (se)*	Std. B (se)	*p*
(Intercept)	im	-2.20 (0.35)	0 (0.03)	**<.001**	iy	68.68 (3.56)	0 (0.03)	**<.001**
High Psychopathy > Controls	a	1.56 (0.15)	0.38 (0.04)	**<.001**	c'	34.19 (1.61)	0.66 (0.03)	**<.001**
logk					b	0.91 (0.37)	0.07 (0.03)	**.01**
Age		-0.02 (0.01)	-0.12 (0.04)	**.001**		-0.08 (0.07)	-0.04 (0.03)	.19
Gender (Male > Female/Other)		0.12 (0.14)	0.03 (0.03)	**.04**		3.91 (1.37)	0.06 (0.03)	**.02**
Income		-0.07 (0.03)	-0.07 (0.03)	.05		-0.70 (0.33)	-0.06 (0.03)	**.04**
Fluid Intelligence		0.10 (0.03)	0.10 (0.03)	**.005**		-0.54 (0.34)	-0.04 (0.03)	.11
	Adjusted R^2 = 0.191				Adjusted R^2 = 0.50			

Note. The value of the indirect effect was *b* = 1.42, *p* = .018; the value of the direct effect (path c) was *b* = 34.19, *p* < .001 and the value of the total effect was *b* = 35.62, p < .001. logk partially mediated the relationship between psychopathy group and STAB score with the proportion mediated being *b* = 0.04 (*p* = .018). *p* < .05 in bold

Table S13

logk Does Not Mediate the Relationship Between Psychopathic Traits and Antisocial Behavior

Variable	logk (M)				STAB (Y)			
	path	*b (se)*	Std. B (se)	*p*	path	*b (se)*	Std. B (se)	*p*
(Intercept)	im	-3.41 (0.40)	0 (0.03)	**<.001**	iy	31.19 (3.59)	0 (0.02)	**<.001**
Psychopathic Traits	a	0.022 (0.002)	0.40 (0.04)	**<.001**	c'	0.55 (0.02)	0.81 (0.03)	**<.001**
logk					b	0.16 (0.31)	0.01 (0.03)	.62
Age		-0.02 (0.01)	-0.10 (0.04)	**.01**		0.06 (0.06)	0.03 (0.02)	.26
Gender (Male > Female/Other)		-0.04 (0.14)	-0.01 (0.03)	.76		-0.82 (1.16)	-0.02 (0.02)	.48
Income		-0.07 (0.03)	-0.08 (0.03)	**.03**		-1.06 (0.28)	-0.09 (0.02)	**<.001**
Fluid Intelligence		0.10 (0.03)	0.10 (0.03)	**.003**		-0.22 (0.29)	-0.02 (0.02)	.45
	Adjusted R^2 = 0.16				Adjusted R^2 = 0.64			

Note: The value of the indirect effect was *b* = 0.003, *p* = .67; the value of the direct effect (path c) was *b* = .55, *p* < .001 and the value of the total effect was *b* = .56, *p* < .001. logk did not mediate the relationship between psychopathic traits and antisocial behavior (STAB). *p* < .05 in bold

Table S14

Interaction Between Psychopathy Group and logk Predicts Antisocial Behavior (STAB)

Variable	*b (se)*	CI	Std. B *(se)*	Std. CI	*p*
(Intercept)	65.99 (3.65)	58.83, 73.1 6	-0.04 (0.03)	-0.09, 0.02	**<.00 1**
High Psychopathy > Controls	38.06 (2.05)	34.04, 42.0 8	0.64 (0.03)	0.58, 0.70	**<.00 1**
logk	-0.03 (0.48)	-0.97, 0.92	0.07 (0.03)	0.01, 0.13	.96
Age	-0.09 (0.06)	-0.22, 0.03	-0.04 (0.03)	-0.09, 0.01	.15
Male	3.56 (1.37)	0.87, 6.25	0.07 (0.03)	0.02, 0.12	**.01**
Income	-0.70 (0.33)	-1.35, -0.05	-0.06 (0.03)	-0.11, -0.004	**.03**
Fluid Intelligence	-0.47 (0.34)	-1.14, 0.20	-0.04 (0.03)	-0.09, 0.02	.17
High Psychopathy > Controls × logk	2.27 (0.75)	0.80, 3.73	0.09 (0.03)	0.03, 0.15	**.002**
	F(7,707) = 105.5, p < .001, Adjusted R^2 = 0.51				

Note. p < .05 in bold

Table S15

Interaction Between Psychopathic Traits and logk Predicts Antisocial Behavior (STAB)

Variable	*b (se)*	CI	Std. B *(se)*	Std. CI	*p*
(Intercept)	30.97 (3.79)	23.54, 38.41	0.00 (0.02)	-0.05, 0.05	**<.001**
Psychopathic Traits (TriPM)	0.56 (0.02)	0.51, 0.59	0.81 (0.03)	0.76, 0.86	**<.001**
logk	0.03 (0.75)	-1.43, 1.49	0.01 (0.03)	-0.04, 0.06	.97
Age	0.06 (0.06)	-0.05, 0.17	0.03 (0.02)	-0.02, 0.08	.27
Male	-0.79 (1.16)	-3.08, 1.49	-0.02 (0.02)	-0.06, 0.03	.49
Income	-1.06 (0.28)	-1.61, -0.51	-0.09 (0.02)	-0.13, -0.04	**<.001**
Fluid Intelligence	-0.22 (0.29)	-0.78, 0.35	-0.02 (0.02)	-0.06, 0.03	.46
Psychopathic Traits × logk	0.00 (0.01)	-0.01, 0.02	0.00 (0.02)	-0.04, 0.05	.85
	$F(7,707) = 186.9$, $p < .001$, Adjusted $R^2 = 0.65$				

Note. p < .05 in bold

Table S16

No Interaction Between Psychopathy Group and Age in Predicting logk

Variable	*b (se)*	CI	Std. B *(se)*	Std. CI	*p*
(Intercept)	-2.03 (0.39)	-2.79, -1.27	0.02 (0.04)	-0.06, 0.09	**<.001**
High Psychopathy > Controls	1.07 (0.51)	0.07, 2.07	0.39 (0.04)	0.31, 0.46	**.04**
Age	-0.03 (0.01)	-0.04, -0.01	-0.11 (0.04)	-0.19, -0.04	**.001**
Male	0.12 (0.14)	-0.15, 0.39	0.03 (0.03)	-0.04, 0.10	.37
Income	-0.07 (0.03)	-0.14, -0.004	-0.07 (0.03)	-0.14, -0.004	**.04**
Fluid Intelligence	0.10 (0.03)	0.03, 0.16	0.10 (0.03)	0.03, 0.17	**.005**
High Psychopathy > Controls × Age	0.01 (0.01)	-0.01, 0.04	0.04 (0.04)	-0.04, 0.12	.31
	$F(6,708) = 29.02$, $p < .001$, Adjusted $R^2 = 0.191$				

Note. p < .05 in bold

Table S17

No Interaction Between Psychopathic Traits and Age in Predicting logk

Variable	*b (se)*	CI	Std. B *(se)*	Std. CI	*p*
(Intercept)	-2.98 (0.64)	-4.24, -1.72	0.01 (0.04)	-0.06, 0.09	**<.001**
Psychopathic Traits (TriPM)	0.02 (0.01)	0.003, 0.03	0.40 (0.04)	0.33, 0.48	**.01**
Age	-0.03 (0.01)	-0.06, -0.001	-0.10 (0.04)	-0.17, -0.02	**.04**
Male	-0.05 (0.14)	-0.32, 0.23	-0.01 (0.03)	-0.08, 0.06	.74
Income	-0.08 (0.03)	-0.14, -0.01	-0.08 (0.03)	-0.15, -0.01	**.02**
Fluid Intelligence	0.10 (0.03)	0.03, 0.1	0.10 (0.03)	0.03, 0.17	**.003**
Psychopathic Traits × Age	0.00 (0.00)	-0.0002, 0.0005	0.03 (0.04)	-0.04, 0.11	.39
	F(6,708) = 30.83, p < .001, Adjusted R2 = 0.20				

Note. p < .05 in bold

Below are supplemental tables for the AUC analyses where participants in the high psychopathy group who were below the 95th percentile cutoff for their gender were transferred to controls and controls above the cutoff were transferred to the high psychopathy group

Table S18

Psychopathy Group Predicting AUC

Variable	b (se)	CI	Std. B *(se)*	Std. CI	p
(Intercept)	0.38 (0.06)	0.26, 0.50	0.00 (0.04)	-0.07, 0.07	**<.001**
High Psychopathy > Controls	-0.19 (0.03)	-0.25, -0.14	-0.27 (0.04)	-0.35, -0.19	**<.001**
Age	0.00 (0.00)	0.001, 0.01	0.09 (0.04)	0.02, 0.17	**.02**
Gender (Male > Female/Other)	-0.05 (0.03)	-0.10, -0.003	-0.07 (0.04)	-0.14, -0.005	**.04**
Income	0.01 (0.01)	-0.005, 0.02	0.04 (0.04)	-0.03, 0.11	.25
Fluid Intelligence	-0.02 (0.01)	-0.03, -0.01	-0.12 (0.04)	-0.19, -0.05	**.001**
	$F(5,709) = 18.21$, $p < .001$, Adjusted $R^2 = 0.108$				

Note. p < .05 in bold

Figure S3

Psychopathic Traits Predicting AUC

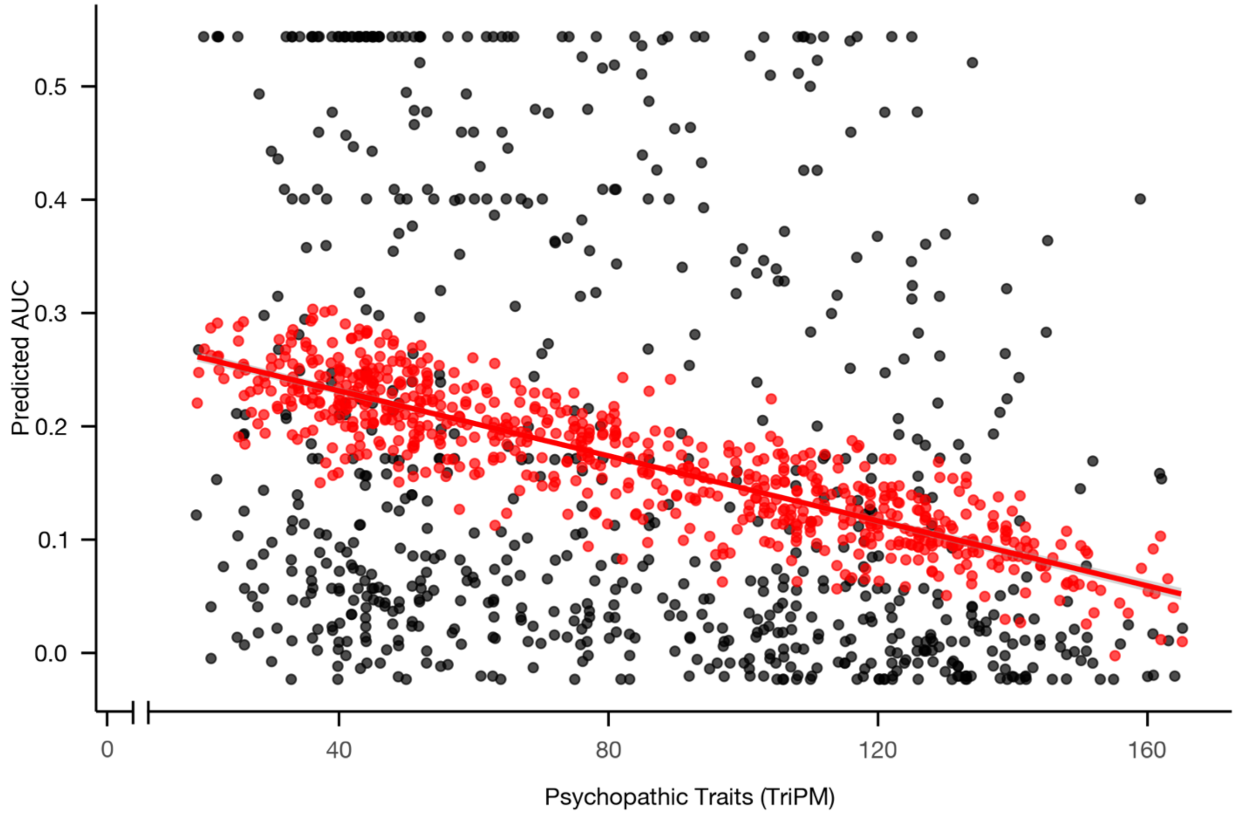

Note. The relationship between psychopathic traits and area under the curve with black representing raw AUC values, and red representing predicted AUC values from the linear regression model controlling for age, gender, fluid intelligence, and income.

Table S19

Psychopathic Traits Predicting AUC

Variable	b (se)	CI	Std. B *(se)*	Std. CI	p
(Intercept)	0.50 (0.07)	0.35, 0.64	0.00 (0.04)	-0.07, 0.07	**<.001**
Psychopathy (TriPM)	0.00 (0.00)	-0.003, -0.002	-0.26 (0.04)	-0.34, -0.18	**<.001**
Age	0.00 (0.00)	0.0003, 0.005	0.09 (0.04)	0.01, 0.16	**.03**
Gender (Male > Female/Other)	-0.03 (0.03)	-0.08, 0.02	-0.05 (0.04)	-0.12, 0.02	.19
Income	0.01 (0.01)	-0.005, 0.02	0.04 (0.04)	-0.03, 0.12	.22
Fluid Intelligence	-0.02 (0.01)	-0.03, -0.01	-0.12 (0.04)	-0.19, -0.05	**.001**
	$F(5,709) = 17.06$, $p < .001$, Adjusted $R^2 = 0.101$				

Note. $p < .05$ in bold

Table S20

Meanness Predicting AUC

Variable	b (se)	CI	Std. B *(se)*	Std. CI	p
(Intercept)	0.42 (0.07)	0.28, 0.57	0.00 (0.03)	-0.07, 0.07	**<.001**
Meanness (TriPM)	-0.01 (0.00)	-0.01, -0.01	-0.43 (0.07)	-0.56, -0.30	**<.001**
Disinhibition (TriPM)	0.00 (0.00)	-0.001, 0.004	0.09 (0.06)	-0.02, 0.20	.12
Boldness (TriPM)	0.00 (0.00)	-0.0001, 0.005	0.08 (0.04)	-0.01, 0.17	.07
Age	0.00 (0.00)	-0.001, 0.004	0.06 (0.04)	-0.02, 0.13	.14
Male > Female/Other	-0.03 (0.02)	-0.08, 0.02	-0.05 (0.04)	-0.12, 0.02	.19
Income	0.01 (0.01)	-0.01, 0.02	0.03 (0.04)	-0.04, 0.11	.36
Fluid Intelligence	-0.02 (0.01)	-0.03, -0.01	-0.12 (0.04)	-0.19, -0.05	**.001**
	F(7,707) = 16.46, p < .001, Adjusted R^2 = 0.13				

Note. p < .05 in bold

Table S21

AUC Does Not Mediate the Relationship Between Psychopathy Group and Antisocial Behavior

Variable	AUC (M)				STAB (Y)			
	path	*b (se)*	Std. B *(se)*	*p*	path	*b (se)*	Std. B *(se)*	*p*
(Intercept)	im	0.38 (0.06)	0 (0.04)	**<.001**	iy	67.69 (3.56)	0 (0.03)	**<.001**
High Psychopathy > Controls	a	-0.19 (0.03)	-0.27 (0.04)	**<.001**	c'	35.11 (1.56)	0.67 (0.03)	**<.001**
AUC					b	-2.66 (2.07)	-0.04 (0.03)	.20
Age		0.003 (0.001)	0.09 (0.04)	**.02**		-0.10 (0.06)	-0.04 (0.03)	.14
Gender (Male > Female/Other)		-0.05 (0.03)	-0.07 (0.04)	**.04**		3.17 (1.38)	0.06 (0.03)	**.02**
Income		0.007 (0.006)	0.04 (0.04)	.252		-0.74 (0.33)	-0.06 (0.03)	**.03**
Fluid Intelligence		-0.02 (0.006)	-0.12 (0.04)	**.001**		-0.51 (0.34)	-0.04 (0.03)	.14
	Adjusted R2 = 0.108				Adjusted R2 = 0.497			

Note. The value of the indirect effect was $b = 0.51$, $p = .21$; the value of the direct effect (path c) was $b = 35.11$, $p < .001$ and the value of the total effect was b = 35.62, $p < .001$. AUC did not mediate the relationship between psychopathy group and STAB score. $p < .05$ in bold

Table S22

AUC Does Not Mediate the Relationship Between Psychopathic Traits and Antisocial Behavior

Variable	AUC (M)				STAB (Y)			
	path	*b (se)*	Std. B *(se)*	*p*	path	*b (se)*	Std. B *(se)*	*p*
(Intercept)	im	0.50 (0.07)	0 (0.04)	**<.001**	iy	31.27 (3.50)	0 (0.02)	**<.001**
Psychopathic Traits	a	-0.002 (0.0004)	-0.26 (0.04)	**<.001**	c'	0.55 (0.02)	0.81 (0.03)	**<.001**
AUC					b	-1.22 (1.73)	-0.02 (0.02)	.48
Age		0.003 (0.001)	0.09 (0.04)	**.03**		0.06 (0.06)	0.03 (0.02)	.26
Gender (Male > Female/Other)		-0.03 (0.03)	-0.05 (0.04)	.19		-0.86 (1.16)	-0.02 (0.02)	.46
Income		0.007 (0.006)	0.04 (0.04)	.22		-1.06 (0.28)	-0.09 (0.02)	**<.001**
Fluid Intelligence		-0.02 (0.006)	-0.11 (0.04)	**.001**		-0.23 (0.29)	-0.02 (0.02)	.43
	Adjusted R^2 = 0.101				Adjusted R^2 = 0.646			

Note: The value of the indirect effect was *b* = 0.003, *p* = .53; the value of the direct effect (path c) was *b* = 0.55, *p* < .001 and the value of the total effect was *b* = 0.56, *p* < .001. AUC did not mediate the relationship between psychopathic traits and antisocial behavior (STAB). *p* < .05 in bold

Figure S4

AUC Predicting Antisocial Behavior in High Psychopathy and Controls

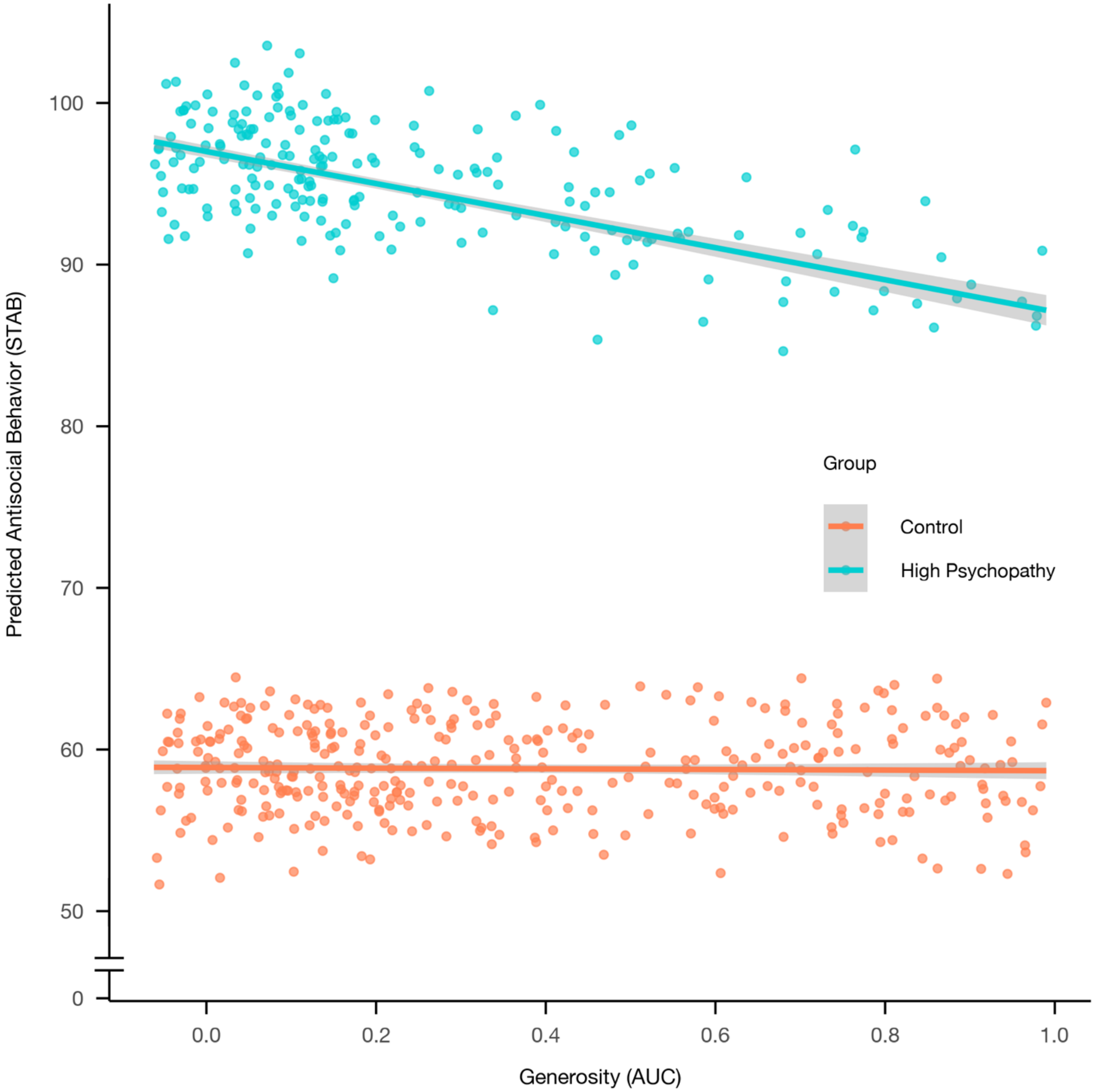

Note. This figure shows predicted antisocial behavior values from the regression model as a function of AUC for each psychopathy group. As AUC increases, antisocial behavior decreases for the high psychopathy group, but not controls.

Table S23

Interaction Between Psychopathy Group and AUC Predicting Antisocial Behavior (STAB)

Variable	b (se)	CI	Std. B *(se)*	Std. CI	p
(Intercept)	63.41 (3.58)	59.48, 73.56	-0.02 (0.03)	-0.07, 0.03	<.001
High Psychopathy > Controls	37.35 (1.86)	33.86, 41.21	0.66 (0.03)	0.60, 0.72	<.001
AUC	-1.90 (2.44)	-4.32, 5.36	-0.05 (0.03)	-0.11, 0.01	.83
Age	-0.05 (0.06)	-0.23, 0.03	-0.05 (0.03)	-0.10, 0.01	.11
Male	1.89 (1.38)	0.61, 6.03	0.06 (0.03)	0.01, 0.12	.02
Income	-0.18 (0.33)	-1.39, -0.09	-0.06 (0.03)	-0.11, -0.01	.02
Fluid Intelligence	-0.44 (0.34)	-1.16, 0.19	-0.04 (0.03)	-0.09, 0.02	.16
High Psychopathy > Controls × AUC	-9.24 (4.46)	-19.07, -1.69	-0.07 (0.03)	-0.13, -0.01	.02
	$F(7,707) = 103.2$, $p < .001$, Adjusted $R^2 = 0.50$				

Note. p < .05 in bold

Table S24

Interaction Between Psychopathic Traits and AUC Predicting Antisocial Behavior (STAB)

Variable	b (se)	CI	Std. B *(se)*	Std. CI	p
(Intercept)	29.78 (3.59)	24.37, 38.57	0.00 (0.02)	-0.04, 0.05	<.001
Psychopathic Traits (TriPM)	0.57 (0.02)	0.51, 0.59	0.81 (0.03)	0.76, 0.87	<.001
AUC	7.10 (4.03)	-10.07, 5.72	-0.02 (0.02)	-0.06, 0.03	.59
Age	0.02 (0.05)	-0.04, 0.17	0.03 (0.02)	-0.02, 0.08	.25
Gender (Male > Female/Other)	-0.68 (1.16)	-3.16, 1.40	-0.02 (0.02)	-0.06, 0.03	.45
Income	-0.38 (0.28)	-1.61, -0.51	-0.09 (0.02)	-0.13, -0.04	<.001
Fluid Intelligence	-0.41 (0.28)	-0.79, 0.34	-0.02 (0.02)	-0.06, 0.03	.43
Psychopathic Traits × AUC	-0.11 (0.05)	-0.08, 0.11	0.01 (0.02)	-0.04, 0.06	.79
	$F(7,707) = 187$, $p < .001$, Adjusted $R^2 = 0.646$				

Note. $p < .05$ in bold

Table S25

No Interaction Between Psychopathy Group and Age in Predicting AUC

Variable	b (se)	CI	Std. B *(se)*	Std. CI	p
(Intercept)	0.35 (0.07)	0.21, 0.49	-0.02 (0.04)	-0.09, 0.06	<.001
High Psychopathy > Controls	-0.10 (0.09)	-0.28, 0.08	-0.28 (0.04)	-0.36, -0.20	.28
Age	0.00 (0.00)	0.001, 0.01	0.08 (0.04)	0.002, 0.16	.01
Male	-0.05 (0.03)	-0.10, -0.003	-0.07 (0.04)	-0.14, -0.005	.036
Income	0.01 (0.01)	-0.004, 0.02	0.04 (0.04)	-0.03, 0.11	.22
Fluid Intelligence	-0.02 (0.01)	-0.03, -0.01	-0.12 (0.04)	-0.19, -0.05	.001
High Psychopathy > Controls × Age	0.00 (0.00)	-0.01, 0.002	-0.04 (0.04)	-0.13, 0.04	.29
	$F(6,708) = 15.37$, $p < .001$, Adjusted $R^2 = 0.108$				

Note. p < .05 in bold

Table S26

No Interaction Between Psychopathic Traits and Age in Predicting AUC

Variable	b (se)	CI	Std. B *(se)*	Std. CI	p
(Intercept)	0.39 (0.12)	0.16, 0.62	-0.02 (0.04)	-0.10, 0.06	.001
Psychopathic Traits (TriPM)	0 (0)	-0.003, 0.001	-0.27 (0.04)	-0.35, -0.19	.38
Age	0.01 (0)	0.0004, 0.01	0.08 (0.04)	-0.003, 0.15	.04
Male	-0.03 (0.03)	-0.08, 0.02	-0.05 (0.04)	-0.11, 0.02	.2
Income	0.01 (0.01)	-0.004, 0.02	0.05 (0.04)	-0.02, 0.12	.18
Fluid Intelligence	-0.02 (0.01)	-0.03, -0.01	-0.12 (0.04)	-0.19, -0.05	.001
Psychopathic Traits × Age	0.00 (0.00)	-0.0001, 0.00	-0.05 (0.04)	-0.13, 0.03	.23
	$F(6,708) = 14.48$, $p < .001$, Adjusted $R^2 = 0.102$				

Note. p < .05 in bold

The following are the results excluding participants who were outside the cutoff for their recruited group.

Table S27. *Characteristics of High Psychopathy and Control Participants*

	Control	High Psychopathy	*p*-value
**N**	349	278	
**Age (SD), range**	41.20 (11.30), 20-77	31.23 (8.80), 18-68	**< .001**
**Gender**			**.008**
Male	161 (46.13%)	120 (43.17%)	
Female	182 (52.15%)	140 (50.36%)	
Other	6 (1.72%)	18 (6.47%)	
**Education**			**<.001**
High School or equivalent	39 (11.17%)	64 (23.02%)	
Some College	67 (19.20%)	102 (36.69%)	
College degree	184 (52.72%)	86 (30.94%)	
Graduate degree	59 (16.91%)	26 (9.35%)	
**Household Income**			.03
Under $25,000	40 (11.46%)	36 (12.95%)	
$25-49,999	98 (28.08%)	63 (22.66%)	
$50-74,999	84 (24.07%)	55 (19.78%)	
$75-99,999	51 (14.61%)	29 (10.43%)	
$100-124,999	22 (6.30%)	26 (9.35%)	
$125-149,999	15 (4.30%)	9 (3.24%)	
$150-174,999	13 (3.72%)	19 (6.83%)	
Over $175,000	23 (6.59%)	30 (10.79%)	
Don't Know	3 (0.86%)	11 (3.97%)	
**Race**			.54
White, non-Hispanic	222 (63.61%)	175 (62.95%)	
Black/African American, non-Hispanic	28 (8.02%)	15 (5.39%)	
Hispanic	49 (14.04%)	45 (16.19%)	
Other	50 (14.33%)	43 (15.47%)	

Note. In all models, those who selected “Don’t know” for Income were recoded to the mean income bracket of the sample. p-values were obtained with a two-tailed t-test for continuous variables and a chi-squared test for categorical variables. $p < .05$ in bold

Table S28

Descriptive Statistics of Sample without Transfers

	Full Sample				Control (*n* = 349)				High Psychopathy (*n* = 278)			
Variable	*M (SD)*	Range	Skewness	Kurtosis	*M (SD)*	Range	Skewness	Kurtosis	*M (SD)*	Range	Skewness	Kurtosis
Total Psychopathy (TriPM)	83.30 (39.29)	19 - 165	0.19	1.65	51.85 (17.65)	19 - 104	0.65	2.96	122.78 (16.93)	91 - 165	0.28	2.54
Meanness	24.17 (17.16)	0 - 57	0.19	1.64	10.89 (8.53)	0 - 37	0.84	3.01	40.84 (8.53)	17 - 57	-0.27	2.40
Boldness	33.62 (12.05)	2 - 57	-0.30	2.43	26.93 (10.25)	2 - 53	-0.11	2.67	42.01 (8.31)	14 - 57	-0.60	2.97
Disinhibition	25.51 (16.16)	0 - 58	0.17	1.74	14.03 (9.86)	0 - 44	0.87	3.19	39.92 (9.68)	8 - 58	-0.44	2.66
STAB	73.89 (26.15)	32 - 155	0.45	2.42	57.05 (18.01)	32 - 138	1.55	6.51	95.02 (18.24)	52 - 155	0.44	3.15
Fluid IQ	5.37 (2.00)	0 - 13	0.680	3.470	5.50 (2.04)	1 - 13	0.81	3.65	5.21 (1.94)	0 - 10	0.48	3.04
AUC	0.28 (0.34)	-0.06 - 0.99	0.95	2.52	0.38 (0.36)	-0.06 - 0.99	0.59	1.84	0.17 (0.28)	-0.06 - 0.99	1.56	4.53
logk	-1.96 (2.00)	-5.71 - 1.75	-0.16	2.32	-2.75 (1.80)	-5.71 - 1.34	0.00	2.38	-0.98 (1.80)	-5.39 - 1.75	-0.54	2.86

Table S29

Criminal History in Order of Prevalence of Crimes Committed in the High Psychopathy Group

	Control (*n* = 349)			High Psychopathy (*n* = 278)		
	Committed	*Charged*	*Convicted*	*Committed*	*Charged*	*Convicted*
Any	133 (38.11%)	68 (19.48%)	60 (17.19%)	239 (85.97%)	101 (36.33%)	85 (30.58%)
Drug Possession	57 (16.33%)	13 (3.72%)	14 (4.01%)	181 (65.11%)	43 (15.47%)	33 (11.87%)
DUI	60 (17.19%)	19 (5.44%)	23 (6.59%)	174 (62.59%)	28 (10.07%)	27 (9.71%)
Reckless Driving	47 (13.47%)	13 (3.72%)	15 (4.30%)	168 (60.43%)	31 (11.15%)	22 (7.91%)
Vandalism	31 (8.88%)	3 (0.86%)	1 (0.29%)	158 (56.83%)	12 (4.32%)	12 (4.32%)
Larceny	32 (9.17%)	11 (3.15%)	9 (2.58%)	153 (55.04%)	22 (7.91%)	15 (5.40%)
Assault	16 (4.58%)	11 (3.15%)	7 (2.01%)	120 (43.17%)	31 (11.15%)	20 (7.19%)
Intent to Sell Drugs	21 (6.02%)	5 (1.43%)	2 (0.57%)	111 (39.93%)	14 (5.04%)	11 (3.96%)
Truancy	27 (7.74%)	3 (0.86%)	1 (0.29%)	105 (37.77%)	14 (5.04%)	12 (4.32%)
Weapons Possessions	10 (2.87%)	3 (0.86%)	4 (1.15%)	93 (33.45%)	0	10 (3.6%)
Running Away	17 (4.87%)	5 (1.43%)	4 (1.15%)	86 (30.94%)	16 (5.76%)	13 (4.68%)
Burglary	7 (2.01%)	2 (0.57%)	3 (0.86%)	80 (28.78%)	7 (2.52%)	6 (2.16%)
Arson	2 (0.57%)	1 (0.29%)	1 (0.29%)	58 (20.86%)	5 (1.8%)	2 (0.72%)
Robbery	6 (1.72%)	6 (1.72%)	5 (1.43%)	55 (19.78%)	8 (2.88%)	6 (2.16%)
Prostitution	2 (0.57%)	0 (0.00%)	2 (0.57%)	49 (17.63%)	1 (0.36%)	1 (0.36%)
Autotheft	4 (1.15%)	4 (1.15%)	5 (1.43%)	26 (9.35%)	5 (1.8%)	5 (1.8%)
Rape	2 (0.57%)	4 (1.15%)	5 (1.43%)	13 (4.68%)	0	0
Murder	3 (0.86%)	1 (0.29%)	3 (0.86%)	5 (1.80%)	2 (0.72%)	0
**Gun Violence**						
Shoot	3 (0.86%)			15 (5.4%)		
Rob	3 (0.86%)			14 (5.04%)		
Gang	4 (1.15%)			13 (4.68%)		
Kill	1 (0.29%)			9 (3.24%)		
Carjack	6 (1.72%)			1 (0.36%)		

Table S30

Hyperbolic Model Predicting Maximum Willing to Forgo for each Social Distance in High Psychopathy and Control Group

Variable	*b*	*SE*	CI	*p*
(Intercept)	86.58	0.79	85.04, 88.12	**<.001**
logk, mean discounting rate for controls	-2.26	0.42	-3.09, -1.44	**<.001**
High Psychopathy > Controls	1.63	0.18	1.27, 1.98	**<.001**
Age	-0.02	0.01	-0.03, -0.002	**.02**
Gender (Male > Female/Other)	-0.003	0.16	-0.32, 0.32	.99
Household Income	-0.03	0.04	-0.10, 0.05	.52
Fluid Intelligence	0.06	0.04	-0.02, 0.14	.13

Note. $p < .05$ in bold

Table S31

Psychopathy Group Predicting logk

Variable	*b (se)*	CI	Std. B *(se)*	Std. CI	*p*
(Intercept)	-2.26 (0.37)	-2.99, -1.53	0.00 (0.04)	-0.07, 0.07	**<.001**
High Psychopathy > Controls	1.63 (0.16)	1.30, 1.94	0.40 (0.04)	0.33, 0.48	**<.001**
Age	-0.02 (0.01)	-0.03, -0.004	-0.10 (0.04)	-0.18, -0.02	**.01**
Gender (Male > Female/Other)	-0.003 (0.15)	-0.29, 0.28	0.00 (0.04)	-0.07, 0.07	.99
Income	-0.03 (0.04)	-0.10, 0.04	-0.03 (0.04)	-0.10, 0.05	.47
Fluid Intelligence	0.06 (0.04)	-0.01, 0.13	0.06 (0.04)	-0.01, 0.13	.09
	$F(5,621) = 32.64$, $p < .001$, Adjusted $R^2 = 0.20$				

Note. p < .05 in bold

Table S32

Psychopathic Traits Predicting logk

Variable	*b (se)*	CI	Std. B *(se)*	Std. CI	*p*
(Intercept)	-3.26 (0.43)	-4.10, -2.42	0.00 (0.04)	-0.07, 0.07	**<.001**
Psychopathic Traits (TriPM)	0.02 (0.00)	0.016, 0.024	0.41 (0.04)	0.33, 0.49	**<.001**
Age	-0.02 (0.01)	-0.03, -0.005	-0.10 (0.04)	-0.18, -0.03	**.01**
Gender (Male > Female and Other)	-0.17 (0.14)	-0.45, 0.12	-0.04 (0.04)	-0.11, 0.03	.25
Income	-0.03 (0.04)	-0.10, 0.04	-0.03 (0.04)	-0.11, 0.04	.37
Fluid Intelligence	0.08 (0.04)	0.01, 0.15	0.08 (0.04)	0.01, 0.15	**.03**
	F(5,621) = 33.42, p < .001, Adjusted R^2 = 0.21				

Note. p < .05 in bold

Table S33

Psychopathy Group Predicting AUC

Variable	*b (se)*	CI	Std. B *(se)*	Std. CI	*p*
(Intercept)	0.39 (0.07)	0.26, 0.53	0.00 (0.04)	-0.07, 0.07	**<.001**
High Psychopathy > Controls	-0.19 (0.03)	-0.25, -0.14	-0.28 (0.04)	-0.37, -0.19	**<.001**
Age	0.002 (0.00)	-0.0004, 0.005	0.07 (0.04)	-0.01, 0.15	.10
Gender (Male > Female/Other)	-0.04 (0.03)	-0.09, 0.01	-0.05 (0.04)	-0.13, 0.02	.16
Income	-0.003 (0.01)	-0.02, 0.01	-0.02 (0.04)	-0.09, 0.06	.66
Fluid Intelligence	-0.01 (0.01)	-0.03, -0.002	-0.08 (0.04)	-0.16, -0.01	**.03**
	$F(5,621) = 15.37$, $p < .001$, Adjusted $R^2 = 0.103$				

Note. p < .05 in bold

Table S34

Psychopathic Traits Predicting AUC

Variable	*b (se)*	CI	Std. B *(se)*	Std. CI	*p*
(Intercept)	0.48 (0.08)	0.32, 0.63	0.00 (0.04)	-0.07, 0.07	**<.001**
Psychopathic Traits (TriPM)	-0.002 (0.00)	-0.003, -0.002	-0.26 (0.04)	-0.34, -0.17	**<.001**
Age	0.003 (0.00)	0.0001, 0.005	0.09 (0.04)	0.002, 0.17	.05
Gender (Male > Female/Other)	-0.02 (0.03)	-0.07, 0.03	-0.03 (0.04)	-0.10, 0.05	.49
Income	-0.003 (0.01)	-0.02, 0.01	-0.02 (0.04)	-0.09, 0.06	.68
Fluid Intelligence	-0.02 (0.01)	-0.03, -0.003	-0.09 (0.04)	-0.17, -0.02	**.02**
	$F(5,621) = 13.60$, $p < .001$, Adjusted $R^2 = 0.091$				

Note. $p < .05$ in bold

Table S35

Meanness and Disinhibition Predicting Social Discounting Rate (logK)

Variable	*b (se)*	CI	Std. B *(se)*	Std. CI	*p*
(Intercept)	-2.88 (0.43)	-3.73, -2.03	0.00 (0.04)	-0.07, 0.07	**<.001**
Meanness	0.05 (0.01)	0.04, 0.07	0.44 (0.07)	0.30, 0.58	**<.001**
Disinhibition	0.01 (0.01)	-0.01, 0.02	0.04 (0.06)	-0.08, 0.16	.509
Boldness	-0.01 (0.01)	-0.02, 0.01	-0.04 (0.05)	-0.13, 0.04	.32
Age	-0.01 (0.01)	-0.03, 0.001	-0.07 (0.04)	-0.15, 0.005	.07
Gender (Male > Female and Other)	-0.15 (0.14)	-0.43, 0.13	-0.04 (0.04)	-0.11, 0.03	.30
Income	-0.02 (0.04)	-0.09, 0.05	-0.02 (0.04)	-0.09, 0.05	.60
Fluid Intelligence	0.08 (0.04)	0.004, 0.15	0.08 (0.04)	0.004, 0.15	**.04**
	$F(7,619) = 27.31$, $p < .001$, Adjusted $R^2 = 0.23$				

Note. $p < .05$ in bold

Table S36

Meanness Predicting AUC

Variable	*b (se)*	CI	Std. B *(se)*	Std. CI	*p*
(Intercept)	0.41 (0.08)	0.25, 0.56	0.00 (0.04)	-0.07, 0.07	**<.001**
Meanness	-0.01 (0.00)	-0.01, -0.01	-0.42 (0.08)	-0.57, -0.27	**<.001**
Disinhibition	0.001 (0.00)	-0.001, 0.004	0.07 (0.07)	-0.06, 0.20	.30
Boldness	0.003 (0.00)	0, 0.01	0.09 (0.05)	0.0001, 0.19	.05
Age	0.002 (0.00)	-0.001, 0.004	0.05 (0.04)	-0.03, 0.14	.20
Gender (Male > Female and Other)	-0.02 (0.03)	-0.07, 0.03	-0.03 (0.04)	-0.10, 0.05	.44
Income	-0.004 (0.01)	-0.02, 0.01	-0.03 (0.04)	-0.10, 0.05	.51
Fluid Intelligence	-0.02 (0.01)	-0.03, -0.003	-0.09 (0.04)	-0.17, -0.02	**.02**
	$F(7,619) = 12.91$, $p < .001$, Adjusted $R^2 = 0.12$				

Note. p < .05 in bold

Table S37

logk Does Not Mediate the Relationship Between Psychopathy Group and Antisocial Behavior

	logk (M)				STAB (Y)			
Variable	path	*b (se)*	Std. B *(se)*	*p*	path	*b (se)*	Std. B *(se)*	*p*
(Intercept)	im	-2.26 (0.37)	0.00 (0.04)	**<.001**	iy	66.01 (3.86)	0.00 (0.03)	**<.001**
High Psychopathy > Controls	a	1.63 (0.16)	0.40 (0.04)	**<.001**	c'	36.23 (1.75)	0.69 (0.03)	**<.001**
logk					b	0.82 (0.40)	0.06 (0.03)	.05
Age		-0.02 (0.01)	-0.10 (0.04)	**.01**		-0.05 (0.07)	-0.02 (0.03)	.513
Gender (Male > Female/Other)		-0.003 (0.15)	-0.001 (0.04)	.99		3.33 (1.45)	0.06 (0.03)	**.02**
Income		-0.03 (0.04)	-0.03 (0.04)	.47		-0.75 (0.36)	-0.06 (0.03)	**.04**
Fluid Intelligence		0.06 (0.04)	0.06 (0.04)	.09		-0.69 (0.37)	-0.05 (0.03)	.06
	Adjusted R^2 = 0.202				Adjusted R^2 = 0.53			

Note: the value of the indirect effect was b = 1.33, p = .068; the value of the direct effect (path c) was b = 36.23, p < .001 and the value of the total effect was b = 37.56, p < .001. logk did not mediate the relationship between psychopathy group and antisocial behavior. p < .05 in bold

Table S38

logk Does Not Mediate the Relationship Between Psychopathic Traits and Antisocial Behavior

	logk (M)				STAB (Y)			
Variable	path	*b (se)*	Std. B *(se)*	*p*	path	*b (se)*	Std. B *(se)*	*p*
(Intercept)	im	-3.26 (0.43)	0.00 (0.04)	**<.001**	iy	32.04 (3.80)	0.00 (0.02)	**<.001**
Psychopathic Traits	a	0.02 (0.00)	0.41 (0.04)	**<.001**	c'	0.55 (0.02)	0.82 (0.03)	**<.001**
logk					b	0.16 (0.34)	0.01 (0.03)	.64
Age		-0.02 (0.01)	-0.10 (0.04)	**.01**		0.04 (0.06)	0.02 (0.03)	.54
Gender (Male > Female/Other)		-0.17 (0.14)	-0.04 (0.04)	.25		-0.63 (1.23)	-0.01 (0.02)	.61
Income		-0.03 (0.04)	-0.03 (0.04)	.37		-1.10 (0.30)	-0.09 (0.02)	**<.001**
Fluid Intelligence		0.08 (0.04)	0.08 (0.04)	**.03**		-0.13 (0.31)	-0.01 (0.02)	.68
	Adjusted R^2 = 0.206				Adjusted R^2 = 0.666			

Note: The value of the indirect effect was *b* = 0.003, *p* = .68; the value of the direct effect (path c) was *b* = 0.55, *p* < .001 and the value of the total effect was *b* = 0.55, *p* < .001. Logk did not significantly mediate the relationship between psychopathic traits and antisocial behavior (STAB). *p* < .05 in bold

Table S39

AUC Does Not Mediate the Relationship Between Psychopathy Group and Antisocial Behavior

	AUC (M)				STAB (Y)			
Variable	path	*b (se)*	Std. B *(se)*	*p*	path	*b (se)*	Std. B *(se)*	*p*
(Intercept)	im	0.39 (0.07)	0.00 (0.04)	**<.001**	iy	64.73 (3.86)	0.00 (0.03)	**<.001**
High Psychopathy > Controls	a	-0.19 (0.03)	-0.28 (0.04)	**<.001**	c'	37.28 (1.68)	0.71 (0.03)	**<.001**
AUC					b	-1.45 (2.23)	-0.02 (0.03)	.52
Age		0.002 (0.001)	0.07 (0.04)	.10		-0.06 (0.07)	-0.03 (0.03)	.41
Gender (Male > Female/Other)		-0.04 (0.03)	-0.05 (0.04)	.16		3.28 (1.46)	0.06 (0.03)	**.03**
Income		0.00 (0.01)	-0.02 (0.04)	.66		-0.78 (0.36)	-0.06 (0.03)	**.03**
Fluid Intelligence		-0.01 (0.01)	-0.08 (0.04)	**.02**		-0.66 (0.37)	-0.05 (0.03)	.07
	Adjusted R^2 = 0.103				Adjusted R^2 = 0.528			

Note: the value of the indirect effect was *b* = 0.28, *p* = .58; the value of the direct effect (path c) was *b* = 37.28, *p* < .001 and the value of the total effect was *b* = 37.56, *p* < .001. AUC did not mediate the relationship between psychopathy group and antisocial behavior (STAB). *p* < .05 in bold

Table S40

AUC Does Not Mediate the Relationship Between Psychopathic Traits and Antisocial Behavior

	AUC (M)				STAB (Y)			
Variable	path	*b (se)*	Std. B *(se)*	*p*	path	*b (se)*	Std. B *(se)*	*p*
(Intercept)	im	0.48 (0.08)	0.00 (0.04)	**<.001**	iy	31.90 (3.74)	0.00 (0.02)	**<.001**
Psychopathic Traits	a	0.00 (0.00)	-0.26 (0.04)	**<.001**	c'	0.55 (0.02)	0.83 (0.03)	**<.001**
AUC					b	-0.79 (1.86)	-0.01 (0.02)	.67
Age		0.003 (0.00)	0.09 (0.04)	.05		0.04 (0.06)	0.02 (0.03)	.55
Gender (Male > Female/Other)		-0.02 (0.03)	-0.03 (0.04)	.49		-0.67 (1.22)	-0.01 (0.02)	.58
Income		0.00 (0.01)	-0.02 (0.04)	.68		-1.11 (0.30)	-0.09 (0.02)	**<.001**
Fluid Intelligence		-0.02 (0.01)	-0.09 (0.04)	**.02**		-0.13 (0.31)	-0.01 (0.02)	.68
	Adjusted R^2 = 0.091				Adjusted R^2 = 0.666			

Note: the value of the indirect effect was b = 0.002, p = .69; the value of the direct effect (path c) was b = 0.55, p < .001 and the value of the total effect was b = 0.55, p < .001. AUC did not mediate relationship between psychopathic traits and antisocial behavior (STAB). p < .05 in bold

Table S41

Interaction Between Psychopathy Group and logk Predicting Antisocial Behavior (STAB)

Variable	*b (se)*	CI	Std. B *(se)*	Std. CI	*p*
(Intercept)	61.39 (3.94)	53.64, 69.13	-0.06 (0.03)	-0.12, -0.0001	**<.001**
High Psychopathy > Controls	42.32 (2.21)	37.99, 46.65	0.67 (0.03)	0.61, 0.74	**<.001**
logk	-0.76 (0.53)	-1.81, 0.29	0.06 (0.03)	0.002, 0.12	.16
Age	-0.06 (0.07)	-0.19, 0.07	-0.03 (0.03)	-0.09, 0.03	.37
Male	3.92 (1.44)	1.09, 6.74	0.07 (0.03)	0.02, 0.13	**.01**
Income	-0.70 (0.35)	-1.39, -0.004	-0.05 (0.03)	-0.11, -0.0003	.05
Fluid Intelligence	-0.60 (0.36)	-1.31, 0.11	-0.05 (0.03)	-0.10, 0.01	.10
High Psychopathy > Controls × logk	3.53 (0.80)	1.96, 5.10	0.13 (0.03)	0.07, 0.19	**<.001**
	$F(7,619) = 107.8$, $p < .001$, Adjusted $R^2 = 0.54$				

Note. $p < .05$ in bold

Table S42

Interaction Between Psychopathic Traits and logk Predicting Antisocial Behavior (STAB)

Variable	*b (se)*	CI	Std. B *(se)*	Std. CI	*p*
(Intercept)	30.92 (4.00)	23.05, 38.78	-0.01 (0.03)	-0.06, 0.04	**<.001**
Psychopathic Traits (TriPM)	0.56 (0.02)	0.52, 0.60	0.82 (0.03)	0.76, 0.88	**<.001**
logk	-0.48 (0.79)	-2.03, 1.07	0.01 (0.03)	-0.04, 0.06	.54
Age	0.03 (0.06)	-0.09, 0.15	0.01 (0.03)	-0.04, 0.06	.61
Gender (Male > Female/Other)	-0.51 (1.23)	-2.93, 1.91	-0.01 (0.02)	-0.06, 0.04	.68
Income	-1.09 (0.30)	-1.69, -0.49	-0.09 (0.02)	-0.13, -0.04	**<.001**
Fluid Intelligence	-0.12 (0.31)	-0.73, 0.49	-0.01 (0.02)	-0.06, 0.04	.69
Psychopathic Traits × logk	0.01 (0.01)	-0.01, 0.02	0.02 (0.03)	-0.03, 0.07	.37
	$F(7,619) = 179.7$, $p < .001$, Adjusted $R^2 = 0.67$				

Note. p < .05 in bold

Table S43

No Interaction Between Psychopathy Group and AUC Predicting Antisocial Behavior (STAB)

Variable	*b (se)*	CI	Std. B *(se)*	Std. CI	*p*
(Intercept)	31.66 (3.82)	24.16, 39.17	0.00 (0.02)	-0.05, 0.05	**<.001**
High Psychopathy > Controls	0.55 (0.02)	0.51, 0.59	0.82 (0.03)	0.77, 0.88	**<.001**
AUC	0.41 (4.23)	-7.89, 8.71	-0.01 (0.03)	-0.06, 0.04	.92
Age	0.03 (0.06)	-0.08, 0.15	0.01 (0.03)	-0.04, 0.07	.57
Male	-0.65 (1.23)	-3.06, 1.76	-0.01 (0.02)	-0.06, 0.03	.59
Income	-1.11 (0.30)	-1.70, -0.51	-0.09 (0.02)	-0.13, -0.04	**<.001**
Fluid Intelligence	-0.13 (0.31)	-0.74, 0.48	-0.01 (0.02)	-0.06, 0.04	.68
High Psychopathy > Controls × AUC	-0.02 (0.05)	-0.12, 0.08	-0.01 (0.03)	-0.06, 0.04	.75
	$F(7,619) = 179.4$, $p < .001$, Adjusted $R^2 = 0.66$				

Note. $p < .05$ in bold

Table S44

Interaction Between Psychopathic Traits and AUC Predicting Antisocial Behavior (STAB)

Variable	*b (se)*	CI	Std. B *(se)*	Std. CI	*p*
(Intercept)	62.60 (3.86)	55.02, 70.19	-0.04 (0.03)	-0.09, 0.02	**<.001**
Psychopathic Traits (TriPM)	41.31 (1.98)	37.43, 45.19	0.69 (0.03)	0.63, 0.75	**<.001**
AUC	4.34 (2.69)	-0.94, 9.63	-0.05 (0.03)	-0.10, 0.01	.11
Age	-0.07 (0.07)	-0.21, 0.07	-0.03 (0.03)	-0.09, 0.03	.32
Gender (Male > Female/Other)	3.51 (1.45)	0.67, 6.36	0.07 (0.03)	0.01, 0.12	**.02**
Income	-0.73 (0.36)	-1.44, -0.03	-0.06 (0.03)	-0.11, -0.003	**.04**
Fluid Intelligence	-0.63 (0.36)	-1.34, 0.09	-0.05 (0.03)	-0.10, 0.01	.09
Psychopathic Traits × AUC	-17.70 (4.70)	-26.92, -8.48	-0.11 (0.03)	-0.17, -0.06	**<.001**
	$F(7,619) = 105$, $p < .001$, Adjusted $R^2 = 0.54$				

Note. $p < .05$ in bold

Table S45

No Interaction Between Psychopathy Group and Age in Predicting logk

Variable	*b (se)*	CI	Std. B *(se)*	Std. CI	*p*
(Intercept)	-2.05 (0.43)	-2.89, -1.21	0.02 (0.04)	-0.06, 0.10	**<.001**
High Psychopathy > Controls	1.11 (0.54)	0.05, 2.17	0.41 (0.04)	0.33, 0.49	**.04**
Age	-0.02 (0.01)	-0.04, -0.01	-0.09 (0.04)	-0.17, -0.01	**.01**
Male	-0.01 (0.15)	-0.29, 0.28	0.00 (0.04)	-0.07, 0.07	.96
Income	-0.03 (0.04)	-0.10, 0.04	-0.03 (0.04)	-0.10, 0.04	.41
Fluid Intelligence	0.06 (0.04)	-0.01, 0.13	0.06 (0.04)	-0.01, 0.13	.09
High Psychopathy > Controls × Age	0.02 (0.02)	-0.01, 0.04	0.04 (0.04)	-0.04, 0.13	.32
	$F(6,620) = 27.37$, $p < .001$, Adjusted $R^2 = 0.20$				

Note. p < .05 in bold

Table S46

No Interaction Between Psychopathic Traits and Age in Predicting logk

Variable	*b (se)*	CI	Std. B *(se)*	Std. CI	*p*
(Intercept)	-2.83 (0.67)	-4.14, -1.52	0.01 (0.04)	-0.06, 0.09	**<.001**
Psychopathic Traits (TriPM)	0.02 (0.01)	0.003, 0.03	0.42 (0.04)	0.34, 0.50	**.02**
Age	-0.03 (0.02)	-0.06, -0	-0.10 (0.04)	-0.18, -0.01	.05
Male	-0.17 (0.14)	-0.46, 0.11	-0.04 (0.04)	-0.11, 0.03	.24
Income	-0.04 (0.04)	-0.11, 0.04	-0.04 (0.04)	-0.11, 0.04	.32
Fluid Intelligence	0.08 (0.04)	0.01, 0.15	0.08 (0.04)	0.01, 0.15	**.03**
Psychopathic Traits × Age	0.0002 (0.00)	-0.0002, 0.001	0.03 (0.04)	-0.05, 0.11	.40
	$F(6,620) = 27.96$, $p < .001$, Adjusted $R^2 = 0.21$				

Note. $p < .05$ in bold

Table S47

No Interaction Between Psychopathy Group and Age in Predicting AUC

Variable	*b (se)*	CI	Std. B *(se)*	Std. CI	*p*
(Intercept)	0.35 (0.08)	0.19, 0.50	-0.02 (0.04)	-0.11, 0.06	**<.001**
High Psychopathy > Controls	-0.09 (0.10)	-0.28, 0.10	-0.29 (0.04)	-0.38, -0.21	.37
Age	0.003 (0.00)	0.0001, 0.01	0.06 (0.04)	-0.03, 0.14	**.04**
Male	-0.04 (0.03)	-0.09, 0.02	-0.05 (0.04)	-0.13, 0.02	.17
Income	-0.002 (0.01)	-0.01, 0.01	-0.01 (0.04)	-0.09, 0.07	.77
Fluid Intelligence	-0.01 (0.01)	-0.03, -0.002	-0.09 (0.04)	-0.16, -0.01	**.03**
High Psychopathy > Controls × Age	-0.003 (0.00)	-0.01, 0.002	-0.05 (0.04)	-0.14, 0.04	.25
	$F(6, 620) = 13.04$, $p < .001$, Adjusted $R^2 = 0.1034$				

Note. p < .05 in bold

Table S48

No Interaction Between Psychopathic Traits and Age in Predicting AUC

Variable	*b (se)*	CI	Std. B *(se)*	Std. CI	*p*
(Intercept)	0.36 (0.12)	0.12, 0.59	-0.02 (0.04)	-0.11, 0.06	**.004**
Psychopathic Traits (TriPM)	-0.001 (0.00)	-0.003, 0.002	-0.26 (0.04)	-0.35, -0.18	.55
Age	0.01 (0.00)	0.0003, 0.01	0.07 (0.04)	-0.01, 0.16	**.04**
Male	-0.02 (0.03)	-0.07, 0.03	-0.02 (0.04)	-0.10, 0.05	.52
Income	-0.002 (0.01)	-0.01, 0.01	-0.01 (0.04)	-0.09, 0.07	.79
Fluid Intelligence	-0.02 (0.01)	-0.03, -0.003	-0.09 (0.04)	-0.17, -0.02	**.02**
Psychopathic Traits × Age	0.00 (0.00)	-0.0001, 0	-0.06 (0.04)	-0.14, 0.03	.20
	$F(6,620) = 11.63$, $p < .001$, Adjusted $R^2 = 0.09$				

Note. p < .05 in bold

Supplementary References

Berluti, K., Ploe, M. L., Doherty, H., Jones, D. N., Patrick, C. J., & Marsh, A. A. (2025). Prevalence and Correlates of Psychopathy in the General Population. *Journal of personality disorders*, *39*(1), 1–21. https://doi.org/10.1521/pedi.2025.39.1.1

Geisen, E. (2022, August 4). *Using Attention Checks in Your Surveys May Harm Data Quality*. Qualtrics. https://www.qualtrics.com/blog/attention-checks-and-data-quality/

Hibben, K. C., Felderer, B., & Conrad, F. G. (2022). Respondent commitment: Applying techniques from face-to-face interviewing to online collection of employment data. *International Journal of Social Research Methodology*, *25*(1), 15–27. https://doi.org/10.1080/13645579.2020.1826647

Jones, B., & Rachlin, H. (2006). Social Discounting. *Psychological Science*, *17*(4), 283–286. https://doi.org/10.1111/j.1467-9280.2006.01699.x

Kuhn, M. (2008). Building Predictive Models in R Using the caret Package. Journal of Statistical Software, 28(5), 1–26. https://doi.org/10.18637/jss.v028.i05

Tingley D., Yamamoto, T., Hirose, K., Keele, L., Imai, K. (2014). mediation: R Package for Causal Mediation Analysis. Journal of Statistical Software, 59(5), 1-38. URL http://www.jstatsoft.org/v59/i05/.
