# Supplementary material for: Adults with more severe psychopathy in the community show increased social discounting
Source: Commun Psychol. 2025 Nov 26;3:175. doi: 10.1038/s44271-025-00353-z (PMC12658011; doi:10.1038/s44271-025-00353-z)
Supplement: Supplementary file 3 — Reporting Summary [file 44271_2025_353_MOESM3_ESM.pdf]

nature portfolio

Corresponding author(s): Naomi Nero

Last updated by author(s): 10/11/2025

Reporting Summary

Nature Portfolio wishes to improve the reproducibility of the work that we publish. This form provides structure for consistency and transparency in reporting. For further information on Nature Portfolio policies, see our Editorial Policies and the Editorial Policy Checklist.

Statistics

For all statistical analyses, confirm that the following items are present in the figure legend, table legend, main text, or Methods section.

n/a	Confirmed	
☐	☒	The exact sample size (*n*) for each experimental group/condition, given as a discrete number and unit of measurement
☐	☒	A statement on whether measurements were taken from distinct samples or whether the same sample was measured repeatedly
☐	☒	The statistical test(s) used AND whether they are one- or two-sided<br>*Only common tests should be described solely by name; describe more complex techniques in the Methods section.*
☐	☒	A description of all covariates tested
☐	☒	A description of any assumptions or corrections, such as tests of normality and adjustment for multiple comparisons
☐	☒	A full description of the statistical parameters including central tendency (e.g. means) or other basic estimates (e.g. regression coefficient) AND variation (e.g. standard deviation) or associated estimates of uncertainty (e.g. confidence intervals)
☐	☒	For null hypothesis testing, the test statistic (e.g. *F*, *t*, *r*) with confidence intervals, effect sizes, degrees of freedom and *P* value noted<br>*Give P values as exact values whenever suitable.*
☒	☐	For Bayesian analysis, information on the choice of priors and Markov chain Monte Carlo settings
☐	☒	For hierarchical and complex designs, identification of the appropriate level for tests and full reporting of outcomes
☐	☒	Estimates of effect sizes (e.g. Cohen's *d*, Pearson's *r*), indicating how they were calculated

Our web collection on statistics for biologists contains articles on many of the points above.

Software and code

Policy information about availability of computer code

Data collection	Qualtrics was used for data collection
Data analysis	Data analysis was done in R. Data, materials, and analysis code are available at https://doi.org/10.17605/OSF.IO/8DF4N

For manuscripts utilizing custom algorithms or software that are central to the research but not yet described in published literature, software must be made available to editors and reviewers. We strongly encourage code deposition in a community repository (e.g. GitHub). See the Nature Portfolio guidelines for submitting code & software for further information.

Data

Policy information about availability of data

All manuscripts must include a data availability statement. This statement should provide the following information, where applicable:

- Accession codes, unique identifiers, or web links for publicly available datasets
- A description of any restrictions on data availability
- For clinical datasets or third party data, please ensure that the statement adheres to our policy

Data Availability
Data and materials are publicly available at https://doi.org/10.17605/OSF.IO/8DF4N.
Code Availability

Analysis code can be publicly accessed at https://doi.org/10.17605/OSF.IO/8DF4N.

Research involving human participants, their data, or biological material

Policy information about studies with human participants or human data. See also policy information about sex, gender (identity/presentation), and sexual orientation and race, ethnicity and racism.

Reporting on sex and gender	Participant's gender was reported from a self-reported question ("Would you describe yourself as: Female, Male, Nonbinary, Other"). Due to low counts in the nonbinary and other category, both are combined such that reported gender as male, female, and other. In the high psychopathy group (n=288) there were 123 Males (42.71%), 147 Females (51.04%) and 18 who selected other (6.25%). In the control group (n = 427) there were 191 Males (44.73%), 224 Females (52.46%), and 12 who selected other (2.81%). In all analyses, we controlled for gender with Female/Other as the reference group.
Reporting on race, ethnicity, or other socially relevant groupings	Participants self-reported race/ethnicity, household income, and highest education level attained. We report the breakdown of participants in the control and high psychopathy group by race/ethnicity, household income, and education level in Table 1. Counts for race/ethnicity in each group are separated into latino/hispanic, white (non-hispanic), black (non-hispanic) and other. Participants self reported their household income to be within a specific income brackets provided. Lastly, participants specified their highest level of education (high school, some college, college degree, or graduate school). Due to correlations between education level and income as well as the nature of the task involving money, only household income was included as a covariate in all analyses.
Population characteristics	The participants in the high psychopathy group were 18-68 years old (M=31.50) and the participants in the control group were 18-79 years old (M=40.21). Because only age ranges can be pre-specified in CloudResearch the control group was older than the high psychopathy participants. As a result, age was included as a covariate in all models.
Recruitment	This study involved examining group differences between the high psychopathy and control group. Participants were recruited through The Society for the Prevention of Disorders of Aggression website if they intially scored in the 95th percentile or above for their gender on the Triarchic Psychopathy Measure (TriPM). Controls were recruited through CloudResearch. After taking the survey for the current study, those recruited to the high psychopathy group who scored below the 95th percentile cutoff for their gender were moved to the control group, and those recruited to the control group who scored above the cutoff were moved to the high psychopathy group (results were consistent when data was re-analyzed after dropping all reassigned participants; see Supplementary Materials). Our high-psychopathy participants sought information about psychopathy online prior to then being invited to participate in this study. Thus, they were in part self-selected. However, we also found similar results whether including or excluding high-psychopathy CloudResearch panel participants.
Ethics oversight	This study was approved by the Georgetown University Institutional Review Board (ID#: 0000193).

Note that full information on the approval of the study protocol must also be provided in the manuscript.

Field-specific reporting

Please select the one below that is the best fit for your research. If you are not sure, read the appropriate sections before making your selection.

☐ Life sciences ☒ Behavioural & social sciences ☐ Ecological, evolutionary & environmental sciences

For a reference copy of the document with all sections, see nature.com/documents/nr-reporting-summary-flat.pdf

Behavioural & social sciences study design

All studies must disclose on these points even when the disclosure is negative.

Study description	This is a quantitative study that uses a cross-sectional design utilizing a purposive sample.
Research sample	Our recruitment approach yielded a sample diverse several demographic variables including age and gender. This study comprises of a novel, community sample of very-high psychopathy adults (n=288), as well as a sample of demographically similar controls (n=427) from the United States. The study's sample had a large age range with the participants in the high psychopathy group being between 18-68 years old (M=31.50) and the participants in the control group being between 18-79 years old (M=40.21). Each group had a similar distribution of gender represented (high psychopathy - 42.71% Male 51.04% Female, 6.25% Other; control - 44.73% Male, 52.46% Female, 2.81% Other)
Sampling strategy	Participants included a community sample of 366 very high-psychopathy participants recruited through the 501(c)(3) non-profit organization Psychopathy Is (now The Society for the Prevention of Disorders of Aggression, https://www.disordersofaggression.org), which provides information and resources for individuals and families affected by psychopathy and related disorders. Visitors can complete screening tests on the website, including the Triarchic Psychopathy Measure (TriPM; Patrick et al., 2009), a 58-item self-report measure that assesses three psychopathy subscales: boldness, meanness, and disinhibition (to protect visitors' privacy, no data are collected about participants or their scores on this measure by the website). Participants who receive TriPM scores in the top 5% of American adults of their gender (Berluti et al., 2024) receive information about diagnostic and treatment options, as well a link to take part in research upon providing their contact information, age, gender, and country of residence. We invited respondents who were 18 or older and indicated they resided in the United States to participate in this study. A total of 727 participants took part

in this study, a sample size determined using the effect size generated from a recent meta-analysis (Amormino et al., 2024), that found this sample would yield >80% power to identify group differences.

Data collection	Data was collected using Qualtrics. Participants recruited through SPDA were emailed an invitation to participate in this study. Those who confirmed interest were sent the Qualtrics link. A subset of the high psychopathy participants (n = 44; 15%) completed the Psychopathy Checklist Screening Version (PCL-SV) with 2-4 trained interviewers via zoom. Researchers were not blind to the participants' being recruited to the high psychopathy group when interacting with them via email. Control participants were recruited through CloudResearch.
Timing	Data collection with participants recruited through the SPDA website began on 4/27/23 and concluded on 2/25/24. We invited the 1,242 respondents who had provided their email information following the completion of the TriPM on the SPDA website as of 11/13/23, were 18 or older, and indicated they reside in the United States to complete the study. Of these, 464 confirmed interest in participating in this study. We continued online recruitment until we achieved our intended sample of high-psychopathy participants. In addition, 361 control participants were recruited through CloudResearch and were recruited between 1/30/24 and 2/7/24.
Data exclusions	Prior to conducting group-based analyses, 78 participants whose TriPM scores fell below the estimated 95th percentile for their gender (Male=105; F or O=91) were removed from the high-psychopathy group and reassigned to the control group. Cutoffs were derived from percentiles calculated using scores from a quasi-representative sample of U.S. adults who completed the TriPM (Berluti et al., 2024). In addition, 10 controls who scored above the cutoff scores for their gender were reassigned to the high psychopathy group (results were consistent when data was re-analyzed after dropping all reassigned participants; see Supplementary Materials). Finally, 12 participants who failed two or more of four attention checks (n =10 high-psychopathy and 2 controls) were excluded from analysis. Thus, our final sample of 715 included 288 high-psychopathy participants and 427 controls.
Non-participation	Of the initial 1,242 individuals contacted from the SPDA website, 464 responded with interest in completing this study, a 37% response rate. Of those 464 individuals who stated interest in participating and we sent the survey to, 366 completed the survey (79% response rate). Controls were recruited through CloudResearch with the aim of collecting demographically similar participants.
Randomization	Participants were not randomized into groups. However, we control for age, gender, income, and fluid intelligence in all of the statistical analyses.

Reporting for specific materials, systems and methods

We require information from authors about some types of materials, experimental systems and methods used in many studies. Here, indicate whether each material, system or method listed is relevant to your study. If you are not sure if a list item applies to your research, read the appropriate section before selecting a response.

Materials & experimental systems

n/a	Involved in the study	
☒	☐	Antibodies
☒	☐	Eukaryotic cell lines
☒	☐	Palaeontology and archaeology
☒	☐	Animals and other organisms
☒	☐	Clinical data
☒	☐	Dual use research of concern
☒	☐	Plants

Methods

n/a	Involved in the study	
☒	☐	ChIP-seq
☒	☐	Flow cytometry
☒	☐	MRI-based neuroimaging

Plants

Seed stocks	*Report on the source of all seed stocks or other plant material used. If applicable, state the seed stock centre and catalogue number. If plant specimens were collected from the field, describe the collection location, date and sampling procedures.*
Novel plant genotypes	*Describe the methods by which all novel plant genotypes were produced. This includes those generated by transgenic approaches, gene editing, chemical/radiation-based mutagenesis and hybridization. For transgenic lines, describe the transformation method, the number of independent lines analyzed and the generation upon which experiments were performed. For gene-edited lines, describe the editor used, the endogenous sequence targeted for editing, the targeting guide RNA sequence (if applicable) and how the editor was applied.*
Authentication	*Describe any authentication procedures for each seed stock used or novel genotype generated. Describe any experiments used to assess the effect of a mutation and, where applicable, how potential secondary effects (e.g. second site T-DNA insertions, mosiacism, off-target gene editing) were examined.*
